# Pandemic potential of the Nipah virus and public health strategies adopted during outbreaks: Lessons from Kerala, India

**DOI:** 10.1371/journal.pgph.0003926

**Published:** 2024-12-19

**Authors:** Thekkumkara Surendran Anish, Reghukumar Aravind, Chandni Radhakrishnan, Nivedita Gupta, Pragya D. Yadav, Jerin Jose Cherian, Rima Sahay, Shubin Chenayil, Anoop Kumar A. S., Anitha Puduvail Moorkoth, Ashadevi, Velichapat Ramakrishnan Lathika, Shamsudeen Moideen, Sekhar Lukose Kuriakose, Kalathil Joseph Reena, Thomas Mathew

**Affiliations:** 1 Kerala One Health Centre for Nipah Research and Resilience, Kozhikode, Kerala, India; 2 Department of Community Medicine, Government Medical College, Wayanad, Kerala, India; 3 Department of Infectious Diseases, Government Medical College, Thiruvananthapuram, Kerala, India; 4 Department of Internal Medicine, Government Medical College, Kozhikode, Kerala, India; 5 Indian Council of Medical Research, New Delhi, India; 6 Indian Council of Medical Research- National Institute of Virology, Pune, Maharashtra, India; 7 Department of Global Public Health, Karolinska Institutet, Stockholm, Sweden; 8 District Surveillance Officer, Malappuram, Kerala, India; 9 Aster MIMS, General Hospital, Kozhikode, Kerala, India; 10 Department of Microbiology, Government Medical College, Kozhikode, Kerala, India; 11 Department of Health Services, Kozhikode, Kerala, India; 12 IQRAA International Hospital and Research Centre, Kozhikode, Kerala, India; 13 Kerala State Disaster Management Authority, Thiruvananthapuram, Kerala, India; 14 Department of Medical Education, Thiruvananthapuram, Kerala, India; Emory University School of Medicine, UNITED STATES OF AMERICA

## Abstract

Kerala, a south Indian state witnessed several outbreaks of Nipah encephalitis since 2018, a zoonotic viral disease with significant pandemic potential. This review highlights the relevance of surveillance and health system preparedness, infection control, early diagnosis and treatment with broad-spectrum antivirals, environmental conservation, and community engagement in mitigating Nipah outbreaks. Additionally, it emphasises the importance of developing new biologicals and anti-viral drugs to combat the disease. The article discusses the available evidence on the spillover mechanisms, genetic attributes of the circulating virus, ecological factors, risk of hospital-based superspreading, treatment outcomes and successful strategies employed in Kerala in response to the recurrent Nipah outbreaks.

## Introduction

The Indian state of Kerala has witnessed six Nipah virus (NiV) spillovers since 2018, two turned out to be outbreaks with clustering of human cases. The first spillover which turned out to be an outbreak happened in May 2018 and led to 23 (18 confirmed and five probable) infections, of which all except two confirmed individuals died [1]. The most recent outbreak was in September 2023 with six cases and two fatalities [2–5]. Kerala experienced four spillover events (June 2019, September 2021, July 2024 and September 2024) limited to single cases [5–8].

NiV is an emerging high-threat pathogen (HTP) with epidemic potential [9]. This negative-sense RNA Henipavirus of the Paramyxovirus family exhibits rapid mutation [10] and infects diverse hosts [11], raising concerns about potentially enhanced transmission risks for humans, reservoir hosts and intermediate hosts [12, 13]. The genus of Henipavirus was identified in 1994, following an outbreak of Hendravirus (HeV) affecting horses which contracted the infections from the reservoir hosts (bats) in Hendra, Queensland, Australia. There have been multiple outbreaks of HeV infection among horses with significant morbidity and mortality in animals since then. Accidental transmission to humans was also reported because of the close contact with the body fluids of affected animals [14]. The pigs were the intermediate hosts responsible for the first reported outbreak of Nipah among the farmers and butchers of Malaysia and Singapore in 1998–99 affecting more than 250 individuals and causing 100 deaths [14–17]. There were no reported human-to-human transmission and the healthcare providers were spared from infection despite their unprotected patient interactions [15]. The outbreak was supposedly contained by culling millions of pigs, inflicting a heavy economic burden on the affected communities [16]. The epicentre of Nipah encephalitis shifted to Bangladesh and the neighbouring regions of India by 2001, and Kerala from 2018 onwards. No intermediate hosts have been identified for the clade of NiV in Bangladesh and India, and most of the patients contracted the disease through human-to-human spread after the initial spillover [1–4, 6, 7, 18–20]. Superspreading events were reported in Bangladesh and India, and hospitals appeared as epicentres of outbreaks [1–4, 18–20]. There is thought to be a difference in the mechanism of NiV spillover between Malaysia and Indo-Bangladesh. In Malaysia and Australia (for HeV), the animals acting as the intermediate hosts got infections from the bats, and spillover happened to humans through the body fluids of the infected animals (pigs and horses) [14]. But in Bangladesh, it is thought that human behaviours like consuming raw date palm sap resulted in contracting the disease directly from the bats [21]. So, studying bat behaviours, human interactions with potential intermediate hosts, symptomatology of the infection in intermediate hosts and risky behaviours like consumption of raw date palm sap are critical in strategizing the prevention and control of NiV spillovers. Currently, Bangladesh and Kerala are the only locations with active transmission of NiV among humans, and bats of the species *Pteropus medius are* the major reservoir hosts. The Nipah outbreak in the Philippines (2014) indicates that animal-mediated outbreaks can still happen through intermediate hosts like pigs and horses [14, 17, 22].

*Pteropus* bats, the reservoir hosts of NiV are distributed over a wide geospatial terrain of South and Eastern Asia [23], a densely populated area with significant biodiversity [24] and many of the countries located in this region may have a health system requiring better means to detect NiV spillovers if it happens [3]. This review aims to articulate the recent experiential learning from repeated Nipah outbreaks in Kerala to the evidence base of this emerging infectious disease with significant pandemic threats.

### The genome of NiV, immunology, transmission mechanism and clinical presentation

NiV genome has not been studied extensively, but significant deviations were noted in samples from humans and animals from Malaysia, Bangladesh and India [1, 2, 25–29]. The authors agree with the WHO recommendation to avoid naming viruses based on the location they were discovered to avoid fear and stigma [30]. However, in the absence of such a clear-cut classification of NiV based on genetic differences, we are compelled to name the virus as described in the literature. It is thought that there are two clades, clade II identified from Malaysia (NiV-M) and other eastern Asian countries, and clade I isolated from Bangladesh and India (NiV-B) [25, 31, 32]. The genetic characterisation of the variant from human and bat samples of various outbreaks in Kerala since 2018 has led to the classification of a new variant, which is genetically close to the clade I (NiV-I) [1, 2, 28, 29, 33]. Clade I-infected patients in Bangladesh and India carried a significantly higher case fatality rate (CFR) [>70%] than that of clade II-infected patients in Malaysia and Singapore [<40%]. NiV clade II isolated cases led to a pure encephalitic syndrome (neurological symptoms without respiratory manifestations) [15], whereas many patients infected with NiV clade I had added severe pulmonary involvement and higher chances of human-to-human transmission [1, 2, 19, 20]. Animal studies demonstrated that clade I is more pathogenic, resulting in an increased viral load in secretions, a shorter incubation period, and a higher mortality rate [34–36].

The nucleotide and amino acid identity of the isolate from the 2018 outbreak in Kerala [NiV-I] was about 97% and 95% with the clade I sequence from Bangladesh and about 91% and 83% with the clade II sequences from Malaysia. The sequence consists of a total of 746 mutations, with 560 being synonymous and 187 non-synonymous. Within these, the Indian clade has 56 specific mutations, of which 31 are non-synonymous and 24 are synonymous [29]. Genetic sequencing of human derived NiV clade I samples in Bangladesh derived to distinct, geographically and temporarily intermingled sub-clades: Bangladesh 1 and Bangladesh 2 [37]. The Bangladesh 1 clade contains 38 specific mutations, with 30 being synonymous and 8 non-synonymous, while the Bangladesh 2 clade has 5 specific mutations, consisting of 4 synonymous and 1 non-synonymous mutation. Additionally, there are 10 mutations present in all clades, comprising 3 non-synonymous and 7 synonymous mutations. The mutations within different Nipah sequences are summarized in the S1 Table.

Mohandas et al, demonstrated that intra-peritoneal infection of the Indian isolate in a 10- to 12-week-old Syrian hamster model resulted in dose-dependent multi-organ disease with significant vascular changes and impairment in the lungs, brain and kidney, and extravascular lesions in the brain and lung [29]. Congestion, haemorrhages, infiltration of inflammatory cells, thrombosis, and, on rare occasions, endothelial syncytial cell development were observed in the blood vessels. The intranasal infection led to a respiratory tract illness with the symptoms of pneumonia. The animal model exhibited clinical characteristics identical to human NiV infection, except for myocarditis. The alteration discovered in NiV I genomes at the amino acid region should be investigated deeper to understand potential significance.

The virus enters the host cells through Ephrin B2/B3 receptors [38–41]. The structure and function of these receptors in humans and animals may make them prone to NiV infection [40]. NiV’s inherent ability to use the evolutionarily conserved and widely expressed cellular receptor Ephrin B2 for entry, a broad range of host species tropism, and its ability to achieve high virus titres make NiV highly transmissible and a pandemic threat [38, 39]. It may be noted that NiV infection could be experimentally induced in many animals which include syrian hamsters, mice, cats, dogs, nonhuman primates, horses, and pigs [42]. There was no significant difference in the receptor function between species indicating the possibility of many intermediate hosts for Nipah [43].

Structural and functional similarities of these receptors across diverse species capable of acting as intermediate hosts in endemic countries are worth studying. The physiological differences of these receptors among humans and their role in contracting the disease are unknown. A keen observation of the Closed Circuit Television (CCTV) footage of hospitals where the superspreading events occurred in Kerala during the 2018 and 2023 outbreaks revealed that some individuals with close unprotected interactions with the infected persons were spared from getting infected. However, certain other individuals in the superspreading location without close interaction with the index case also got infected. The differential transmission among the contacts necessitates further study on host factors related to infection susceptibility, including immunological responses, cellular receptor tropisms and affinity, which make certain people more vulnerable to the infection. Similar to the bat-mediated rabies transmitted through aerosols, the possibility of infected bats or intermediate hosts directly infecting individuals with favourable immunological attributes, is worth exploring [44–48]. From the visuals of the locations of superspreading in Kerala (CCTV footage), we realised that the infected healthcare providers were not using masks at the time of interaction with the patients, and all patients and caregivers who wore masks were protected despite closer interaction, as reported previously [49]. However, all unprotected interactions did not end up in infections necessitating more studies on the transmission mechanism of Nipah. Moreover, we noticed that transient contacts or proximity to the superspreading primary case ended up in infections on a few occasions. The morphological and functional differences of the Ephrin B2/B3 receptors among the human population are worth exploring.

In Bangladesh and India, many people were infected through super-spreading events at hospitals. Such super-spreading events could act as the engine of a pandemic if they occur widely and limit the health system’s ability to contain outbreaks [50, 51]. To our best knowledge, NiV is transmitted only through exposure to body fluids, contaminated materials or respiratory droplets and only amongst people in close contact [20]. But just like SARS-CoV-2, the threat of NiV acquiring favourable mutations over the years enabling its transmission through air is a concern to be addressed [46]. This is the reason why the World Health Organization [WHO] included NiV in the list of priority pathogens/ agents and called for global investment towards research and development [9, 52].

### Surveillance for reservoirs and hosts

In the context of pandemic threats, surveillance of the natural reservoirs and hosts of NiV is critical. Moreover, such a pre-emptive surveillance mechanism will equip the health system to recognize other viruses and even the entry of other high-risk pathogens like Ebola virus (species orthoebolavirus zairense) and corona viruses including the SARS-Co-V2. NiV is not lethal to bats and those infected do not show any evident manifestations [53]. Hence the virus can’t be eradicated from nature and may accumulate mutations. Southern and eastern parts of Asia, northern Australia and the east part of the African continents constitute the home of fruit bats under the genus *Pteropus* (family- Pteropodidae) [54–56]. The findings of Gokhale et. al. raise questions regarding whether bat genera other than *Pteropus*, like *Rousettus* (family- Pteropodidae) and *Pipistrelles* (family- Vespertilionidae) can act as reservoirs for NiV [54]. There is a hypothesis that the differences in circulating clades in Eastern Asia and Southeast Asia could be attributed to the reservoir hosts in the environment [25]. There is no evidence that the reservoir hosts of HeV, *Pteropus alecto*, *Pteropus conspicillatus*, *Pteropus poliocephalus* and *Pteropus scapulatus* are reservoirs for NiV [57, 58]. However, the close similarity of NiV with HeV, the presence of *Pteropus* bats in the environment, and the detection of antibodies against NiV in other species of *Pteropus* in neighbouring countries put northern Australia at risk of NiV spillovers [59, 60]. Table 1 summarises bat species, their distribution, and their roles in NiV transmission.

**Table 1:** Bat Species with confirmed (presence of RNA and antibody) or suspected (presence of antibody, not RNA) role in NiV transmission and regional distribution.

Bat Species	Confirmed/ Suspected Role in NiV Transmission	Virus clade	Countries at risk
*Pteropus medius* (Indian flying fox) [54, 55, 61]	Confirmed	NiV Clade I	Bangladesh, India, Bhutan, Nepal, Pakistan, Sri Lanka, China, Maldives and Madagascar
*Pteropus vampyrus* (large flying fox) [59, 62, 63]	Confirmed	NiV Clade II	Malaysia, Singapore, Cambodia, China, Indonesia, Myanmar, Philippines, Thailand, Timor-Leste and Vietnam
*Pteropus hypomelanus* (Island flying fox) [64, 65]	Confirmed		
*Pteropus lylei* (Lyle’s flying fox) [66–69]	Confirmed	More close to NiV Clade I	Cambodia, Thailand, Vietnam and China
*Rousettus leschenaultii* [54, 70]	Suspected	NA	Bangladesh, India, Bhutan, Nepal, Pakistan and Sri Lanka
*Pipistrellus spp*. [54, 70]	Suspected	NA	
*Hipoposiderous larvatus* [69]	Suspected	NA	Bangladesh, China, Thailand, Cambodia, Laos, Vietnam, Indonesia, Malaysia, Mynamar, and India

Since 2018, the state of Kerala which is more than 2,000 kilometres away from Bangladesh and the north-eastern Indian state of West Bengal (the initial epicentre of Nipah in India during 2001–2007) has been reporting NiV spillovers by a clade genetically close to that found in Bangladesh [1, 2]. Moreover, the genetic analysis of samples extracted from both the patients and the reservoirs in Kerala showed that the virus might have been in the local environment for a significantly longer time sufficient to aggregate the genetic mutations which make it distinct [28]. All investigations following the previous outbreaks demonstrated a seroprevalence of 9–28% among the *Pteropus* bats in the vicinity, with a serial increase in seroprevalence from 9% in February to 28% in September [54, 61, 71]. Out of the six spillover events reported in Kerala, three happened in September.

Along with saliva and urine samples, we extracted viral RNA from visceral organs and rectal swabs of *P*.*medius* [61, 71]. So, not only the fruits and beverages contaminated by the bat’s saliva or urine, but the exposure to internal organs or excrement of an infected bat is also a concern. Free-ranging domestic animals like cats that predate bats and interact with humans have the potential to act as intermediate hosts [72]. Experimental NiV infection produced neurological and respiratory symptoms in cats and a high amount of NiV RNA was retrieved from the body fluids of the infected animal [73, 74]. Moreover, there is a recent discovery that domestic cats are prone to infection with another paramyxovirus, Feline Morbillivirus (FeMV), and the immunological reaction to the infection is similar to that of NiV and HeV [75]. A seroprevalence study from six spillover sites in Bangladesh also demonstrated a high seroprevalence among cats (0%-20%) compared to other domestic animals [76]. So, the possibility of domestic animals including cats acting as intermediate hosts needs to be explored thoroughly.

### Spillover mechanism

**Fig 1:**
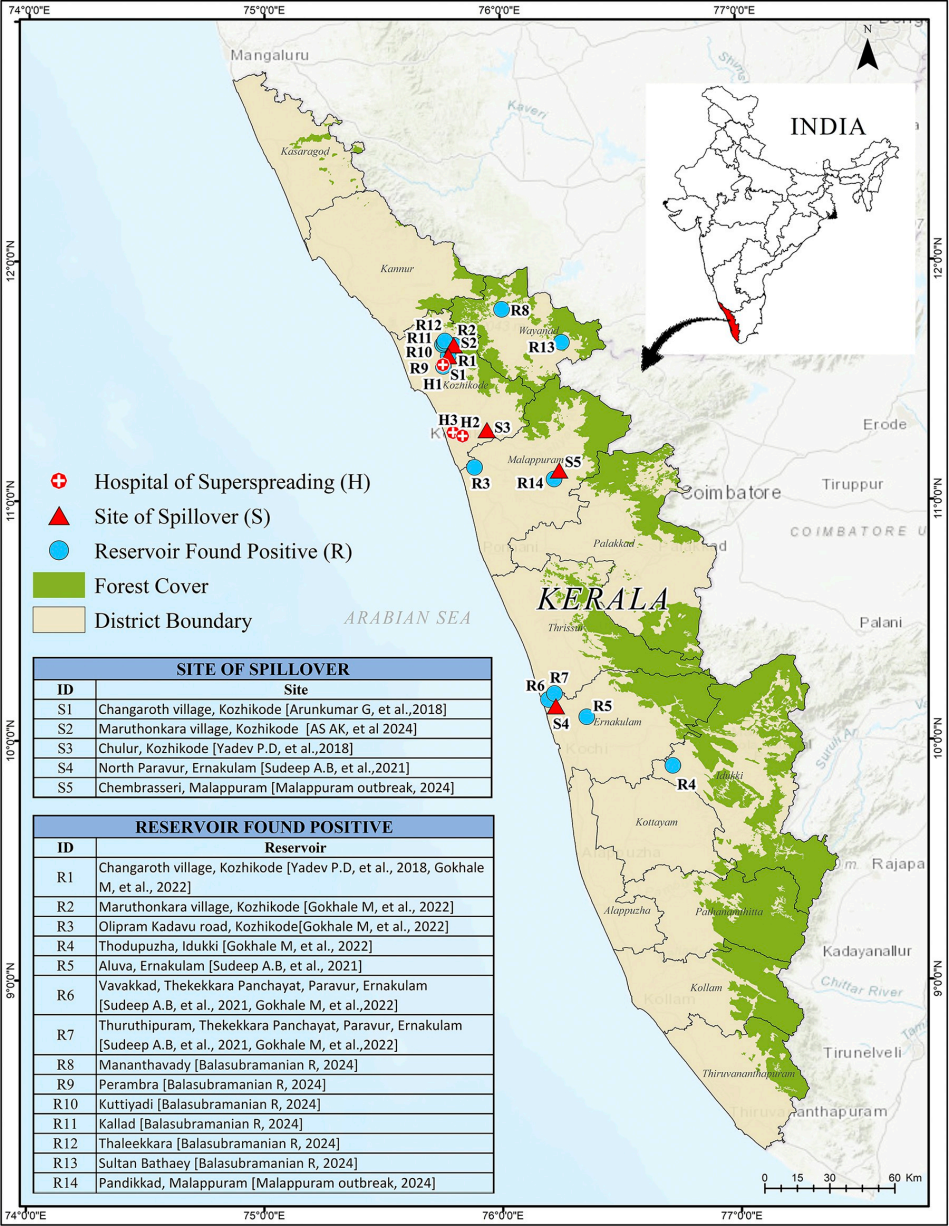
Map showing the locations in the state of Kerala of reported spillovers, hospital-based super spreading events and reservoirs found positive in bats for NiV. Published under a CC BY license with permission from Kerala State Disaster Management Authority (KSDMA), original copyright [2024].

Fig 1 shows the sites of spillovers [1–8] and hospital-based superspreading events (two events in 2018 and one in 2023) [1, 2]. Surveys conducted by the Indian Council of Medical Research (ICMR)-National Institute of Virology (NIV), Pune in the vicinity of the outbreaks revealed the presence of serological or virological confirmation of the virus in 14 sites from five different districts of Kerala (Fig 1– Kozhikode-R1, R2 and R9 to R12, Malappuram-R3 and R14, Idukki-R4, Ernakulam- R5 to R7 and Wayanad R8 and R13) [54, 61, 71].

Most of the cases reported in Kerala [27/33 (81·8%) of total and 23/28 (82·1%) of RTPCR confirmed] could be attributed to the human-to-human spread. As Kerala has experienced only six spillovers, there is no scope for a case-control study to analyse the behavioural risk factors leading to spillovers. We conducted virological examinations of bitten fruits (since date palm sap is not collected in Kerala), which yielded negative results. However, as shown in Fig 1, we found multiple roosts of *Pteropus medius* around the site of spillovers and all of these roosts reported virological or serological positivity for NiV. In the absence of known risk factors, Kerala followed a comprehensive approach to reduce the chances of spillovers, including educating people not to consume fallen/bitten fruits and bat meat, avoiding consuming nectar in banana flowers, washing banana leaves before food materials are served on them, washing hands with soap and water in case of any accidental exposure to bats or its body fluids [3]. Similar public education and awareness campaigns should be undertaken in other places with geographical proximity to the epicentres, where the Pteropus bats are widely present irrespective of the history of a proven spillover because spillovers of NiV could be missed easily [3].

**Fig 2:**
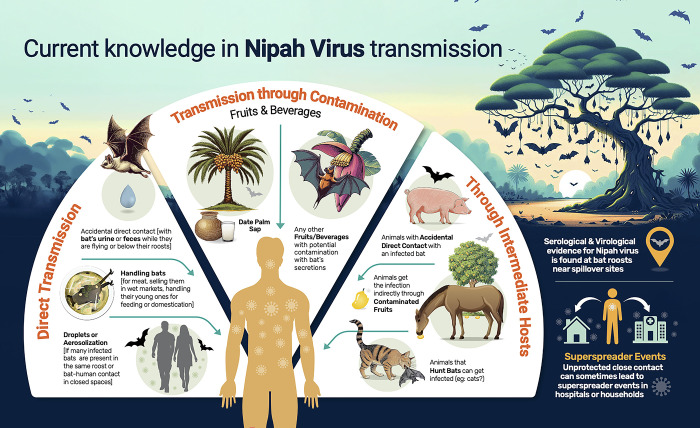
Current knowledge about Nipah virus transmission. Published under a CC BY license with permission from the designer, original copyright [2024].

Transmission through the blood and flesh of infected, and often symptomatic animals (pigs) was demonstrated in Nipah outbreaks in Malaysia [15]. The same thing happened with horses in the Philippines for NiV, and in Australia for HeV [22, 77]. The presence of NiV RNA was also demonstrated in the internal organs of *Pteropus* bats in Kerala [61, 71] and so, handling bat meat could facilitate the spillover similar to that with an infected pig or horse [78]. Some epidemiological studies revealed that drinking unpasteurized date palm sap is a risk factor for Nipah spillovers in Bangladesh, but the viral RNA was never retrieved from the samples [19, 21, 56, 79]. However, this information helped to reduce the number of NiV spillovers there [79–82]. It is likely that naturally available sugary drinks like nectars, fruits or nuts with fleshy carp are being contaminated by body secretions of the bat just like in the case of date palm sap. Infectious materials were isolated from the droppings of bats, but never from fruits, nectars or drinks at the point of consumption [56]. Risk factors other than the consumption of raw date palm sap may be there in Bangladesh, yet to be found out [83]. Even if the initial spillover occurred from date palm sap, any other contaminated object, or from an intermediate host, human-to-human transmission was the trigger of outbreaks in Bangladesh and India. Nipah may transmit through multiple ways simultaneously. Current knowledge about Nipah virus transmission dynamics from bats to humans is given in Fig 2.

### Hospital-acquired Nipah infections

Most clade I cases contracted the disease through human-to-human transmission from a super-spreader infected from the natural reservoir host. Suboptimal infection control measures and limited isolation facilities make hospitals fertile soil for NiV transmission [18]. During Nipah outbreaks in Kerala, all secondary cases other than the family members of the primary contracted the infection from hospitals [1, 2]. This avoidable spread of hospital infection far outnumbered the spillovers and infection among family contacts. The spread of infection happened only from a highly symptomatic patient, during the later course of the illness when the patient developed respiratory distress with encephalitis (Table 2). During this phase of illness, the viral load is likely to be very high and patients start to expel it through their respiratory secretions and body fluids. Hospital-based clusters are likely to originate from such highly infectious and sick patients. Two primary/index cases with respiratory symptoms induced 25 new infections (Table 2) [1, 2]. In four instances out of the six spillovers, we could isolate the patient even before the onset of respiratory manifestations (in 2019, 2021 and two times in 2024) and ended up with zero transmissions [4, 6–8]. Most of the transmission happened from the patient thought to have contracted the infection directly from the reservoir host. However, rare occasions of secondary transmissions were reported (Table 2). The case fatality ratio (CFR), the Effective Reproduction Numbers (Re) and some hypotheses that emerged from Nipah spillovers/outbreaks in Kerala are listed in Table 2. Most of the infections do not end up in transmission but there are occasional superspreading events. To put it differently, even if the mean Re is low, the overdispersion of the distribution of Re makes the infection more prone to large outbreaks [84].

**Table 2:** Case fatality ratio (CFR), Effective Reproduction Numbers and related hypotheses from Nipah spillovers/outbreaks in Kerala (Both RTPCR confirmed and probable cases were included in the table unless specified otherwise) [1, 2, 6–8, 71, 85].

Attribute	Frequency*	Percentage (%)/ Mean (Range)*	Hypothesis*	Risk of bias*
CFR among primary cases	5/6	83.33%	Case fatality is more in primary cases	The difference is narrow, statistically not significant (Fisher exact test). There may be a delay or absence in diagnosis and starting treatment for a primary case
CFR among RTPCR confirmed primary cases	4/5	80.00%		
CFR among secondary/tertiary cases	21/27	77·80%		
CFR among RTPCR confirmed secondary/tertiary cases	17/23	73·90%		
CFR among patients who did not receive any anti-viral medication	14/14	100.00%	Case fatality among patients receiving anti-viral medication on a compassionate ground is low (statistically significant difference, Fisher exact test p value = 0.013). The findings is in agreement with other contexts with Clade I infection.	There may be a selection bias. Patients with comparatively a milder disease with slow progression may get treated with antiviral drugs
CFR among patients received some anti-viral treatment	12/19	63.16%		
CFR among patients received Ribavirin	6/11	54.54%		
CFR among patients received Remdesivir	0/3	0.0%		
Reproduction number of primary cases	25/6	4.17 (0–20)	Reproduction number of primary cases is high. So the chance of superspreading is high with the primary case. The findings is in agreement with other contexts with Clade I infection.	
Reproduction number of secondary cases	2/25	0·08 (0–1)		There may be a selection bias because the secondary and tertiary cases might have identified and isolated early.
Reproduction number of tertiary cases	0/2	0.00%		
Reproduction number of primary cases not isolated before the start of respiratory symptoms	27/2	13·50 (0–22)	Reproduction number of primary cases not isolated before the start of respiratory symptoms is high. So the chance of superspreading is high with those case	NA
Reproduction number of primary cases isolated before the start of respiratory symptoms	0/4	0.00		

*Studies given as references are the sources of data used in this table

## Early detection and treatment

Even though the numbers are limited, CFR in the recent (2023) outbreak in Kerala compared with the outbreak due to a genotypically similar virus in 2018 appeared to be low [(2/6) vs (21/23)] [1, 2]. Early detection and prompt treatment with nonspecific antiviral drugs during the 2023 outbreak might have resulted in a decreased CFR [2, 3]. Diagnosing Nipah encephalitis at the acute viraemic phase itself is a priority and we could offer supportive care and treatment to help the patient. The process of diagnosis involved many stages. As Nipah is still a rare reason for encephalitis in Kerala, epidemiologically unlinked encephalitis cases will undergo a PCR-based panel of investigations as the first step to exclude Nipah. If these tests are negative, at the second step the samples (throat swab, blood, urine and CSF) will be screened for NiV RNA at a biosafety level (BSL) 2/3 facility or a point of care nucleic acid test (Truenat). If it turned positive, the confirmation will be done at the BSL 4 facility of NiV Pune. We do not use serological tests to diagnose Nipah in an active symptomatic suspect because of its low sensitivity in early phase of the infection, but they are used for serosurvey. There are five PCR facilities and two Truenat facilities in Kerala. The Government of Kerala is planning to deploy Truenat-based point-of-care facilities in and around the epicentres of previous spillover sites for better syndromic surveillance of AES/ARDS. Due the increased availability of Truenat, clinicians are finding it useful as the primary test in spillover districts, especially when there is a high index of suspicion. Clinicians and radiologists are being trained to pick up segmental myoclonus and discrete high intensity lesions in brain MRI in patients with encephalitis which will help in arriving at a diagnosis of Nipah encephalitis [86–88].

Since there are no approved treatment measures and due to the high risk of mortality, broad-spectrum antivirals are used on compassionate grounds even if no officially approved vaccines or medicines specifically against NiV. Ribavirin is considered the antiviral of choice in all Henipa viral infections both in humans and animals and it was found to be effective against HeV infections in horses and NiV infections in humans on several occasions [85, 89–91]. During the 2018 outbreak of NiV in Kerala, ribavirin was recommended for use in patients. However, the initial non-availability resulted in only eight patients receiving it and most succumbed without antiviral treatment [85]. All patients who survived a Nipah infection in Kerala received some form of antiviral treatment during their course of illness (Table 2) [2, 85]. During the 2023 outbreak, remdesivir was recommended on compassionate grounds for early use before the onset of encephalitis, and it is believed that the early initiation may have improved the CFR (Table 2) [2]. Remdesivir is a nucleotide analogue prodrug with broad-spectrum antiviral activity that has been shown to inhibit filovirus, coronavirus, and paramyxovirus replication. In-vitro studies, as well as clade 1 challenge studies in African green monkeys, have shown that remdesivir is effective for the treatment as well as prophylaxis against Nipah virus disease [92–94]. The US Centers for Disease Control and Prevention (CDC) and the European Centre for Disease Prevention and Control (ECDC) recognise the potential role of remdesivir and ribavirin in NiV outbreaks [95, 96]. In 2023, remdesivir was readily available at hospitals because of the COVID-19 pandemic, facilitating its timely administration in patients with NiV infection. So Kerala revised its treatment guidelines for Nipah to include the use of remdesivir on compassionate grounds and it this might have helped to reduce the mortality in 2023 [97]. As we are experiencing a potential benefit of using remdesivir in treating Nipah, the State Medical Board recommended the use of antiviral drugs remdesivir (200mg IV loading dose and 100mg once daily thereafter for 12 days) and favipiravir (1800mg twice daily loading dose and 800mg twice daily thereafter for 13 days) for prophylaxis on compassionate grounds among high-risk primary contacts after a risk-benefit analysis was done. We categorised the high-risk primary contacts into two groups, contacts with the highest risk of exposure (n = 33) were given injectable remdesivir and the others (n = 17) were given oral favipiravir. The effectiveness of the prophylaxis could not be evaluated as there was no transmission from the index case.

m102·4 monoclonal antibodies (mAb) targeting the virus are recommended as the specific management option to prevent severe disease manifestations in high-risk individuals [95]. However, the limited global availability of mAb and the rapid progression of the disease have interfered with its use in outbreaks [98]. Another concern with the use of mAb is its potential utility in only the clade against which it has been prepared. Only phase II/III trials evaluating its efficacy can provide conclusive answers to these concerns. Recognizing its potential for preventing deaths, India and Bangladesh have separately initiated research efforts to develop mAbs tailored to locally circulating NiV strains [99, 100]. The Kerala state health department collected serum samples from the survivors of the Nipah (2023 outbreak) and handed them over to ICMR and other research organisations under the public sector to initiate developing mAb from the locally circulating clade of NiV. It is also important to monitor Nipah survivors continuously because of the rare possibility of relapse and late-onset NiV encephalitis [88, 101]. Investigations suggested that it is the reactivation of infection, not due to an immunological reaction leading to demyelination. We have a case with reactivation of infection in Kerala to whom all samples expect brain biopsy are virologically sterile. Late-onset reactivation encephalitis in Nipah appears to be non-contagious, but it is a concern because of the potential social stigma and significant morbidity/mortality risk associated with it.

Vaccine development remains a crucial area of NiV research, with the Global Alliance for Vaccines and Immunisation (Gavi) leading the clinical trials of many vaccine candidates utilising various platforms [102]. These include mRNA [103], protein subunit (employing HeV protein) [104] and vector vaccine platforms [105, 106]. Due to the high mortality rates of NiV, developing an attenuated vaccine without the risk of reversion to the wild variant is also a challenge [107]. A brief description of vaccine candidates is given in S2 Table.

## Ecological factors

Ecological disruption, including deforestation and human encroachments in biodiversity hotspots, are attributed to the emergence of zoonotic diseases such as NiV [108–110]. Four out of the six spillovers of NiV (2018, 2019, 2023, 2024 July) in Kerala happened in ElNino years. Chua KB, et. al. linked the large outbreak of Nipah in Malaysia in 1998 to the severe drought driven by the El Nino Southern Oscillation (ENSO) event [111]. Latinne A and Mrand S also claim that the sudden and marked climatic alterations triggered by ENSO trigger viral spillovers [112]. Elevated ambient temperatures are known to be an environmental stressor to bats and have been attributed to influence infections, viral load and human-animal interactions [109, 112]. With climate change as a growing concern, health systems in endemic areas should prepare for more frequent and wider NiV spillovers. Interestingly, all six spillovers in Kerala occurred during the hot and humid months of May—September, coinciding with the ripening season for almost all seasonal fruits. These observations characterise the environmental factors influencing human-animal interactions that play a critical role in NiV outbreaks.

Kerala’s experience showed that the panic surrounding a NiV outbreak led to some unhealthy social cohesion. It sometimes spiralled into human-animal conflict and ended up in further destruction of bat habitats. Epidemiological investigations following every outbreak identified that people used to cut down trees roosted by bats and often used crackers to fright them to push them away from their dwelling places. It may result in higher probabilities of man-bat interactions directly or indirectly and may cause migration of infected bats to wider locations [111], at the same time hindering bats from performing their ecological roles such as the dispersal of seeds. The destruction of bat habitats can induce NiV spillovers and is a vicious cycle. Forest exploration and adventurous tourism should respect these rules without disturbing these bat habitats. Another major issue is the macro and microeconomic impact of Nipah spillovers. The fruit market in the State faced a collapse. People fear collecting not only fruits but cash crops like areca nuts and cashew nuts also because of contamination by bats [113, 114]. Even though there is not even a single instance of the virus being reported from exported fruits, fruit exports get affected as an overcorrection measure. The containment measures, even if localised to a specific geographical area, affect the microeconomic activities in the affected areas and contribute to the economic loss.

## Response of the health system

Outbreaks of Nipah in Bangladesh and India manifested as small clusters or single cases with high fatality within a short period (often a few days). There is a high chance of spillover events being missed owing to the sporadic nature of the disease unless the health system is vigilant enough to detect small clusters of encephalitis or unusual deaths. Gaps in virological or serological screening further dampen surveillance systems even after the recognition of clinically high-risk cases. Moreover, the endemic zones of NiV are superimposed with that of many tropical fevers presenting with encephalitis, like Japanese encephalitis [115]. The last case in Kerala was interpreted as a case of Dengue encephalitis because of bleeding manifestation was associated [116]. Experiences of Bangladesh and India indicate that the initial outbreaks in an unprepared country could easily be missed. The currently known initial spillovers in both countries were not detected at the onset of the outbreak, but only after antibodies against NiV were detected in stored samples [18, 117]. The risk perception of the communities towards NiV is very important to prevent spillovers from going undetected in potentially endemic countries.

**Fig 3:**
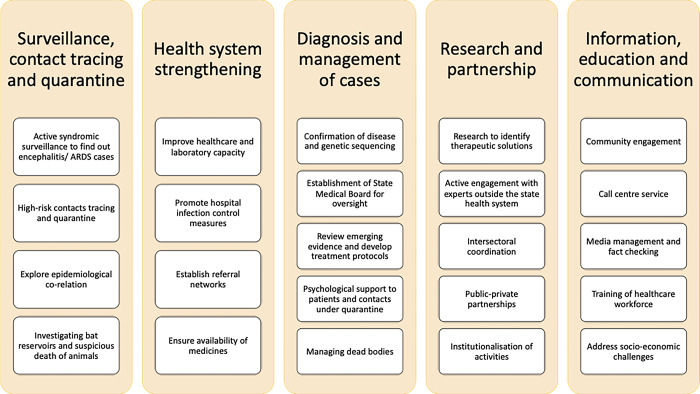
Health system response and activities in Kerala following identification of a NiV spillover.

Kerala’s health system has been improving in its capability to detect a Nipah spillover over the years. The initial three spillovers (2018, 2019 and 2021) were identified by corporate private hospitals. In the case of the latter events (2023, 2024 July and 2024 September) the public health system identified the encephalitis cases from smaller hospitals through epidemiological investigations. A vigilant health system enabled Kerala to conduct virological surveillance in encephalitis/ARDS cases and was instrumental in detecting the outbreak early, even in case of single-case spillovers. The Government of Kerala developed a standard operating procedure (SOP) that helped identify the transmission early and contain the outbreak [97]. Major activities under the health system response in Kerala are summarised in Fig 3.

1. **Surveillance, Contact Tracing, and Quarantine**: To manage the outbreak, comprehensive surveillance was implemented. Community health workers were engaged to identify unreported cases of encephalitis, ARDS, or unusual deaths [118]. A dedicated surveillance team conducted in-depth epidemiological investigations on cases flagged by these workers. Hospitals across the state were directed to screen all suspected cases. High-risk contact tracing involved interviewing patients, friends, and family, using self-reported spatiotemporal maps, GPS tracking, and CCTV footage [97]. Contacts were quarantined at home for 21 days, and monitored by field health workers, and any symptoms were flagged for further action. The contact list was regularly updated and patients with negative tests were excluded from follow-up after 21 days.
2. **Health System Strengthening**: Efforts to strengthen the health system included enhancing existing facilities and establishing new ones. Hospitals were equipped with BSL-2 facilities and strengthened infection control SOPs for healthcare worker safety. A mobile BSL-3 unit was deployed for on-site diagnosis. A dedicated public sector treatment facility was set up in a tertiary hospital to manage additional cases, and regional secondary care hospitals were earmarked for potential outbreaks. Infection control measures were reinforced, including the availability of protective equipment, structural changes where necessary and dedicated ambulances with infection control facilities [119]. Psycho-social support for healthcare workers were provided.
3. **Diagnosis and Management of Cases**: Diagnosis and case management were prioritized to control the outbreak effectively. All hospitals were required to evaluate cases of ILIs, encephalitis, or ARDS for potential links to the outbreak and test them using RTPCR. Use of Truenat Nipah tests helped in faster screening, containment measures and early initiation of management. Laboratory confirmation and genetic sequencing were performed at ICMR-NIV Pune’s BSL-4 facility. Patients who tested positive were isolated at their testing hospitals to prevent further spread. A State Medical Board for Nipah was constituted to oversee the outbreak response and support the government in evidence-guided decision-making. A treatment protocol was developed with the best available evidence and clinicians were trained to adhere to it. Availability of anti-viral medicines were unsured, including the m102·4 mAb that was made available from Queensland University, Australia by efforts of ICMR for the compassionate use in 2018 and 2023.
4. **Research and Partnership**: Research and partnerships played a crucial role in the outbreak response. ICMR-NIV Pune conducted bat studies. Suspicious death of animals in the vicinity were investigated in collaboration with institutions such as the National Institute of High Security Animal Diseases and various experts from veterinary, forestry, and public health fields. Research activities included the production of monoclonal antibodies and environmental detection of viral RNA. Collaboration with national agencies, such as NCDC and ICMR, ensured the integration of emerging evidence into updated treatment protocols [120]. It was critical to ensure public-private partnerships since some patients were admitted in private hospitals and the index cases were detected by the private hospitals at many previous instances. The Kerala One Health Centre for Nipah Research and Resilience (KOHCNRR) was established to focus on research and pandemic preparedness [121].
5. **Information, Education, and Communication**: Effective communication was essential in managing the outbreak. Information about the outbreak and prevention strategies was disseminated through multiple channels, including a dedicated call center available round the clock. The public was educated on how to prevent spillovers and human-to-human transmission. Healthcare workers were trained regarding infection control, treatment protocols, and biosafety. Media communications were centralized to avoid confusion, and misinformation was actively countered with accurate data. Advocacy strategies were adopted to mitigate the socio-economic challenges of the outbreak including stigma of individuals who were infected or under quarantine, and reduced market demand of fruits since it was thought that the disease could spread by consuming them. Psychological support was provided through call center counsellors and psychiatrists. The handling of corpses was carefully managed to address social and religious concerns while preventing transmission [122].

## Conclusion

In conclusion, the eradication of Nipah virus (NiV) remains impractical due to its persistence in natural reservoirs and its tendency to evolve into new variants that may affect a wider range of bat species and intermediate hosts. Climate change further complicates this by potentially driving viral evolution and increasing the risk of human transmission. As human infections impose evolutionary pressure on the virus, it is critical to emphasize early detection and prevention of human-to-human transmission. Addressing modifiable factors contributing to NiV spillovers is essential, and a critical evaluation of Kerala’s public health response can enhance future strategies.

Countries in southern and eastern Asia, northern Australia, and eastern Africa may want to assess their risk and implement evidence-based, risk-informed prevention and control measures. These should include ongoing surveillance of Pteropus bat populations for NiV RNA and antibodies, regular serosurveys of intermediate hosts, and monitoring for Acute Encephalitis Syndrome (AES) and death audits in at-risk human populations. Identifying and modifying human behaviors that contribute to bat interactions and spillovers, such as the consumption of bat-bitten fruits or date palm sap need to be further evaluated.

Additionally, sentinel animals like pigs and horses should undergo serial serosurveys, and alternative intermediate hosts should be investigated in areas with active transmission. Strengthening AES surveillance and deploying point-of-care diagnostic tests will aid in early detection and management of outbreaks. Hospitals in endemic areas must implement strict infection control measures, including dedicated isolation facilities and advanced ventilation systems.

Investments in research for new vaccines, monoclonal antibodies, and antivirals are vital. Continuous monitoring of NiV genomes and clinical manifestations will help assess pandemic potential. Survivors should be monitored for possible reactivation of the virus. A comprehensive one health approach, integrating human-animal interactions, ecological disruption, and climate change, will be crucial for developing resilient strategies against NiV and other high-threat pathogens.

## Supporting information

S1 Table Mutations with different Nipah sequences. (DOCX)

S2 Table Comparison chart of the Nipah virus vaccines under clinical trials. (DOCX)
