# Supplementary material for: Pandemic potential of the Nipah virus and public health strategies adopted during outbreaks: Lessons from Kerala, India
Source: PLOS Glob Public Health. 2024 Dec 19;4(12):e0003926. doi: 10.1371/journal.pgph.0003926 (PMC11658523; doi:10.1371/journal.pgph.0003926)
Supplement: S1 Table — (DOCX) [file pgph.0003926.s001.docx]

**S1 Table:**

**Mutations with different Nipah sequences**

| **Clade in which Mutation present** | **Gene** | **Nucleotide Mutation** | **Amino acid Mutation** | **Malaysia** | | **Cambodia** | | **Thailand** | | **Bangladesh_clade1** | | **Bangladesh_clade2** | | **India** | |
| --- | --- | --- | --- | --- | --- | --- | --- | --- | --- | --- | --- | --- | --- | --- | --- |
|  |  |  |  | **Nucleotide Mutation** | **Amino acid Mutation** | **Nucleotide Mutation** | **Amino acid Mutation** | **Nucleotide Mutation** | **Amino acid Mutation** | **Nucleotide Mutation** | **Amino acid Mutation** | **Nucleotide Mutation** | **Amino acid Mutation** | **Nucleotide Mutation** | **Amino acid Mutation** |
|  | N | C124T |  |  |  |  |  | C124T |  | C124T |  | C124T |  | C124T |  |
|  | N | G169A |  |  |  |  |  | G169A |  | G169A |  | G169A |  | G169A |  |
|  | N | A172G |  |  |  |  |  |  |  |  |  |  |  | A172G |  |
|  | N | A190G |  |  |  |  |  | A190G |  | A190G |  | A190G |  | A190G |  |
|  | N | T199C |  |  |  |  |  |  |  |  |  |  |  | T199C |  |
|  | N | A232G |  |  |  |  |  | A232G |  | A232G |  | A232G |  | A232G |  |
| **Bangladesh clade_Specific** | N | G271A |  |  |  |  |  | G271A |  | G271A |  | G271A |  |  |  |
|  | N | T289C | I59S |  |  |  |  | T289C | I59S | T289C | I59S | T289C | I59S | T289C | I59S |
|  | N | T304A |  |  |  |  |  | T304A |  | T304A |  | T304A |  | T304A |  |
|  | N | C334T |  |  |  | C334T |  | C334T |  | C334T |  | C334T |  | C334T |  |
| **Bangladesh clade_Specific** | N | T382C |  |  |  |  |  | T382C |  |  |  | T382C |  |  |  |
|  | N | A451G |  |  |  | A451G |  | A451G |  | A451G |  | A451G |  | A451G |  |
|  | N | G472A |  |  |  |  |  | G472A |  | G472A |  | G472A |  | G472A |  |
|  | N | C505T |  |  |  |  |  |  |  |  |  |  |  | C505T |  |
| **Present in all clades** | N | A520G |  | A520G |  | A520G |  | A520G |  | A520G |  | A520G |  | A520G |  |
|  | N | A538G |  |  |  | A538G |  | A538G |  | A538G |  | A538G |  | A538G |  |
| **2019_India_specific** | N | G566A | G152S |  |  |  |  |  |  |  |  |  |  | G566A | G152S |
|  | N | C610T |  |  |  |  |  |  |  |  |  |  |  | C610T |  |
|  | N | G625A |  |  |  |  |  | G625A |  | G625A |  | G625A |  | G625A |  |
|  | N | T697C |  |  |  |  |  |  |  |  |  |  |  | T697C |  |
|  | N | G745A | Q211R |  |  |  |  |  |  | G745A | Q211R |  |  |  |  |
|  | N | A760G | E216Q |  |  |  |  | A760G | E216Q | A760G | E216Q | A760G | E216Q | A760G | E216Q |
|  | N | G766A |  |  |  |  |  | G766A |  | G766A |  | G766A |  | G766A |  |
|  | N | C775T | L221V |  |  |  |  | C775T | L221V | C775T | L221V | C775T | L221V | C775T | L221V |
|  | N | C778T | S222C |  |  |  |  | C778T | S222C | C778T | S222C | C778T | S222C | C778T | S222C |
|  | N | A787G |  |  |  |  |  | A787G |  | A787G |  | A787G |  | A787G |  |
|  | N | G796A |  |  |  |  |  | G796A |  | G796A |  | G796A |  | G796A |  |
|  | N | C802T |  |  |  | C802T |  | C802T |  | C802T |  | C802T |  | C802T |  |
|  | N | A820C |  |  |  |  |  | A820C |  | A820C |  | A820C |  | A820C |  |
|  | N | C877T |  |  |  | C877T |  | C877T |  | C877T |  | C877T |  | C877T |  |
|  | N | C967T |  |  |  |  |  | C967T |  | C967T |  | C967T |  | C967T |  |
|  | N | A997G |  |  |  |  |  |  |  | A997G |  |  |  |  |  |
|  | N | A1075G |  |  |  | A1075G |  | A1075G |  | A1075G |  | A1075G |  | A1075G |  |
|  | N | **A1087G** |  |  |  |  |  |  |  |  |  |  |  | A1087G |  |
|  | N | A1096G |  |  |  |  |  | A1096G |  | A1096G |  | A1096G |  | A1096G |  |
|  | N | G1156A |  |  |  | G1156A |  | G1156A |  | G1156A |  | G1156A |  | G1156A |  |
|  | N | T1159C |  |  |  |  |  | T1159C |  | T1159C |  | T1159C |  | T1159C |  |
|  | N | T1171G |  |  |  | T1171G |  |  |  |  |  |  |  | T1171G |  |
|  | N | A1231G |  |  |  |  |  |  |  | A1231G |  |  |  |  |  |
|  | N | T1330C |  |  |  |  |  |  |  |  |  |  |  | T1330C |  |
|  | N | T1333C |  |  |  | T1333C |  | T1333C |  | T1333C |  | T1333C |  | T1333C |  |
|  | N | T1342C |  |  |  |  |  | T1342C |  | T1342C |  | T1342C |  | T1342C |  |
|  | N | A1351T |  |  |  |  |  | A1351T |  | A1351T |  | A1351T |  | A1351T |  |
|  | N | T1378C |  |  |  |  |  | T1378C |  | T1378C |  | T1378C |  | T1378C |  |
|  | N | A1397G | I429V |  |  | A1397G | I429V | A1397G | I429V | A1397G | I429V | A1397G | I429V | A1397G | I429V |
|  | N | G1407A | G432E |  |  | G1407A | G432E | G1407A | G432E | G1407A | G432E | G1407A | G432E | G1407A | G432E |
|  | N | A1433C |  |  |  |  |  | A1433C |  | A1433C |  | A1433C |  | A1433C |  |
|  | N | G1459A |  |  |  |  |  | G1459A |  | G1459A |  | G1459A |  | G1459A |  |
|  | N | A1481G | N457D |  |  | A1481G | N457D | A1481G | N457D | A1481G | N457D | A1481G | N457D | A1481G | N457D |
|  | N | T1501C |  |  |  |  |  |  |  |  |  |  |  | T1501C |  |
|  | N | A1510C |  |  |  |  |  | A1510C |  | A1510C |  | A1510C |  | A1510C |  |
|  | N | C1513A |  |  |  |  |  | C1513A |  | C1513A |  | C1513A |  | C1513A |  |
|  | N | A1537T |  |  |  |  |  | A1537T |  | A1537T |  | A1537T |  | A1537T |  |
|  | N | A1540G |  |  |  |  |  | A1540G |  | A1540G |  | A1540G |  | A1540G |  |
|  | N | A1558G |  |  |  |  |  | A1558G |  | A1558G |  | A1558G |  | A1558G |  |
|  | N | A1567T |  |  |  | A1567T |  | A1567T |  | A1567T |  | A1567T |  | A1567T |  |
|  | N | A1579G |  |  |  |  |  | A1579G |  | A1579G |  | A1579G |  | A1579G |  |
|  | N | C1585T | A491T |  |  |  |  |  |  |  |  |  |  | C1585T | A491T |
|  | N | C1588T |  |  |  |  |  | C1588T |  | C1588T |  | C1588T |  | C1588T |  |
|  | N | A1591G |  |  |  |  |  | A1591G |  | A1591G |  | A1591G |  | A1591G |  |
|  | N | C1594T | S494T |  |  |  |  | C1594T | S494T | C1594T | S494T | C1594T | S494T | C1594T | S494T |
| **India_Specific** | N | G1620A | S503N |  |  |  |  |  |  |  |  |  |  | G1620A | S503N |
| **Bangladesh clade_Specific** | N | G1626A | R505K |  |  |  |  | G1626A | R505K | G1626A | R505K | G1626A | R505K |  |  |
|  | N | A1628G | T506D |  |  |  |  | A1628G | T506D | A1628G | T506D | A1628G | T506D | A1628G | T506D |
|  | N | G1634A | G508R |  |  |  |  | G1634A | G508R | G1634A | G508R | G1634A | G508R | G1634A | G508R |
|  | N | G1645A | E511G |  |  |  |  | G1645A | E511G | G1645A | E511G | G1645A | E511G | G1645A | E511G |
|  | N | T1657C |  |  |  |  |  | T1657C |  | T1657C |  | T1657C |  | T1657C |  |
|  | N | C1663T |  |  |  |  |  | C1663T |  | C1663T |  | C1663T |  | C1663T |  |
| **India_Specific** | N | C1670T | P520S |  |  |  |  |  |  |  |  |  |  | C1670T | P520S |
|  | N | G1673A | A521T |  |  | G1673A | A521T | G1673A | A521T | G1673A | A521T | G1673A | A521T | G1673A | A521T |
|  | N | C1684T |  |  |  |  |  | C1684T |  | C1684T |  | C1684T |  | C1684T |  |
|  | N | T1690C |  |  |  |  |  | T1690C |  | T1690C |  | T1690C |  | T1690C |  |
|  | N | C1693T |  |  |  |  |  | C1693T |  | C1693T |  | C1693T |  | C1693T |  |
|  | P | T2501C |  |  |  | T2501C |  | T2501C |  | T2501C |  | T2501C |  | T2501C |  |
|  | P | T2525C |  |  |  | T2525C |  | T2525C |  | T2525C |  | T2525C |  | T2525C |  |
|  | P | A2527G | Q41R |  |  | A2527G | Q41R | A2527G | Q41R | A2527G | Q41R | A2527G | Q41R | A2527G | Q41R |
|  | P | T2546C |  |  |  |  |  |  |  |  |  |  |  | T2546C |  |
|  | P | C2550T |  |  |  |  |  |  |  |  |  |  |  | C2550T |  |
|  | P | G2586A | G61R |  |  |  |  |  |  | G2586A | G61R |  |  |  |  |
|  | P | T2595C | S64P |  |  |  |  |  |  |  |  | T2595C | S64P |  |  |
| **2023 India_Specific** | P | **G2616A** | **E71K** |  |  |  |  |  |  |  |  |  |  | **G2616A** | **E71K** |
|  | P | A2646G | T81A |  |  |  |  |  |  | A2646G | T81A |  |  |  |  |
| **2023 India_Specific** | P | **T2649C** | **S82P** |  |  |  |  |  |  |  |  |  |  | **T2649C** | **S82P** |
|  | P | T2820C | Y139H |  |  |  |  | T2820C | Y139H | T2820C | Y139H | T2820C | Y139H | T2820C | Y139H |
|  | P | A2823T | T140S |  |  | A2823T | T140S | A2823T | T140S | A2823T | T140S | A2823T | T140S | A2823T | T140S |
|  | P | T2989C | L195P |  |  | T2989C | L195P | T2989C | L195P | T2989C | L195P | T2989C | L195P | T2989C | L195P |
|  | P | T3008C | P201L |  |  |  |  |  |  |  |  |  |  | T3008C | P201L |
|  | P | C3017T |  |  |  |  |  | C3017T |  | C3017T |  | C3017T |  | C3017T |  |
|  | P | T3023C |  |  |  |  |  |  |  |  |  |  |  | T3023C |  |
|  | P | A3047G |  |  |  |  |  |  |  | A3047G |  |  |  |  |  |
|  | P | A3058G | K218R |  |  |  |  | A3058G | K218R | A3058G | K218R | A3058G | K218R | A3058G | K218R |
| **India_Specific** | P | A3061G | E219G |  |  |  |  |  |  |  |  |  |  | A3061G | E219G |
|  | P | G3073A | G223D |  |  |  |  | G3073A | G223D | G3073A | G223D | G3073A | G223D | G3073A | G223D |
|  | P | T3077C |  |  |  |  |  | T3077C |  | T3077C |  | T3077C |  | T3077C |  |
| **India_Specific** | P | C3078G | Q225E |  |  |  |  |  |  |  |  |  |  | C3078G | Q225E |
|  | P | G3085A | S227N |  |  |  |  | G3085A | S227N | G3085A | S227N | G3085A | S227N | G3085A | S227N |
| **Bangladesh clade_Specific** | P | G3088A | R228K |  |  |  |  | G3088A | R228K |  |  | G3088A | R228K |  |  |
| **2023 India_Specific** | P | **T3098C** |  |  |  |  |  |  |  |  |  |  |  | **T3098C** |  |
| **2019_India_specific** | P | A3134G |  |  |  |  |  |  |  |  |  |  |  | A3134G |  |
|  | P | G3137A |  |  |  |  |  |  |  | G3137A |  | G3137A |  | G3137A |  |
|  | P | T3143C |  |  |  |  |  |  |  | T3143C |  |  |  |  |  |
|  | P | T3149C |  |  |  |  |  |  |  | T3149C |  |  |  |  |  |
|  | P | C3155T |  |  |  |  |  | C3155T |  | C3155T |  | C3155T |  | C3155T |  |
|  | P | A3158G |  |  |  |  |  | A3158G |  | A3158G |  | A3158G |  | A3158G |  |
|  | P | A3173G |  |  |  | A3173G |  | A3173G |  | A3173G |  | A3173G |  | A3173G |  |
|  | P | C3176T |  |  |  |  |  | C3176T |  | C3176T |  | C3176T |  | C3176T |  |
|  | P | T3212G | D269E |  |  |  |  | T3212G | D269E | T3212G | D269E | T3212G | D269E | T3212G | D269E |
|  | P | G3218A |  |  |  |  |  |  |  | G3218A |  | G3218A |  | G3218A |  |
|  | P | T3229C | V275A |  |  |  |  | T3229C | V275A | T3229C | V275A | T3229C | V275A | T3229C | V275A |
|  | P | G3234A | G277R |  |  |  |  | G3234A | G277R | G3234A | G277R | G3234A | G277R | G3234A | G277R |
|  | P | C3242T |  |  |  |  |  |  |  | C3242T |  | C3242T |  |  |  |
|  | P | A3244T | N280I |  |  |  |  | A3244T | N280I | A3244T | N280I | A3244T | N280I | A3244T | N280I |
|  | P | C3251T |  |  |  |  |  |  |  | C3251T |  | C3251T |  |  |  |
|  | P | A3252G | I283V |  |  |  |  | A3252G | I283V | A3252G | I283V | A3252G | I283V | A3252G | I283V |
|  | P | G3259A | R285H |  |  | G3259A | R285H |  |  | G3259A | R285H |  |  | G3259A | R285H |
|  | P | C3262T | T286I |  |  |  |  | C3262T | T286I | C3262T | T286I | C3262T | T286I | C3262T | T286I |
| **India_Specific** | P | A3264C | I287L |  |  |  |  |  |  |  |  |  |  | A3264C | I287L |
| **India_2023+Bangladesh Specific** | P | G3267A | E288K |  |  |  |  |  |  | G3267A | E288K | G3267A | E288K | G3267A | E288K |
|  | P | A3275G |  |  |  |  |  |  |  | A3275G |  | A3275G |  |  |  |
|  | P | T3280C | I292T |  |  | T3280C | I292T | T3280C | I292T | T3280C | I292T | T3280C | I292T | T3280C | I292T |
| **2023 India_Specific** | P | **C3282A** | **R293K** |  |  |  |  |  |  |  |  |  |  | **C3282A** | **R293K** |
| **India_2023+Bangladesh Specific** | P | G3283A |  |  |  |  |  |  |  | G3283A |  | G3283A |  | G3283A |  |
|  | P | A3289G | N295S |  |  | A3289G | N295S | A3289G | N295S | A3289G | N295S | A3289G | N295S | A3289G | N295S |
|  | P | T3293C | L296P |  |  | T3293C | L296P | T3293C | L296P | T3293C | L296P | T3293C | L296P | T3293C | L296P |
| **2018 India_Specific** | P | **C3294A** | **Q297K** |  |  |  |  |  |  |  |  |  |  | **C3294A** | **Q297K** |
|  | P | G3297A | A298I |  |  |  |  | G3297A | A298I | G3297A | A298I | G3297A | A298I | G3297A | A298I |
|  | P | A3304G | D300G |  |  | A3304G | D300G | A3304G | D300G | A3304G | D300G | A3304G | D300G | A3304G | D300G |
|  | P | A3311G |  |  |  |  |  | A3311G |  | A3311G |  | A3311G |  | A3311G |  |
|  | P | T3312C | S303P |  |  | T3312C | S303P | T3312C | S303P | T3312C | S303P | T3312C | S303P | T3312C | S303P |
| **Present in all clades** | P | A3315G | T304A | A3315G | T304A | A3315G | T304A | A3315G | T304A | A3315G | T304A | A3315G | T304A | A3315G | T304A |
|  | P | T3322C | V306A |  |  |  |  | T3322C | V306A | T3322C | V306A | T3322C | V306A | T3322C | V306A |
|  | P | C3337T | P311L |  |  |  |  |  |  |  |  |  |  | C3337T | P311L |
|  | P | A3340G | K312R |  |  |  |  |  |  | A3340G | K312R |  |  |  |  |
|  | P | A3347G | S314P |  |  | A3347G | S314P | A3347G | S314P | A3347G | S314P |  |  |  |  |
| **India_2023+Bangladesh Specific** | P | G3353A |  |  |  |  |  |  |  | G3353A |  | G3353A |  | G3353A |  |
|  | P | G3360A | E319K |  |  |  |  | G3360A | E319K | G3360A | E319K | G3360A | E319K | G3360A | E319K |
|  | P | C3363T | P320S |  |  |  |  | C3363T | P320S | C3363T | P320S | C3363T | P320S | C3363T | P320S |
|  | P | A3380G |  |  |  |  |  | A3380G |  | A3380G |  | A3380G |  | A3380G |  |
|  | P | T3392A |  |  |  |  |  | T3392A |  | T3392A |  | T3392A |  | T3392A |  |
|  | P | A3398G |  |  |  |  |  | A3398G |  | A3398G |  | A3398G |  | A3398G |  |
|  | P | G3404A |  |  |  |  |  | G3404A |  | G3404A |  | G3404A |  | G3404A |  |
|  | P | G3416T |  |  |  |  |  | G3416T |  | G3416T |  | G3416T |  | G3416T |  |
|  | P | C3428T |  |  |  |  |  | C3428T |  | C3428T |  | C3428T |  | C3428T |  |
|  | P | T3431C |  |  |  |  |  | T3431C |  | T3431C |  | T3431C |  | T3431C |  |
|  | P | G3433A | R343Q |  |  |  |  | G3433A | R343Q | G3433A | R343Q | G3433A | R343Q | G3433A | R343Q |
|  | P | C3438T |  |  |  |  |  | C3438T |  | C3438T |  | C3438T |  | C3438T |  |
|  | P | G3446A |  |  |  |  |  | G3446A |  | G3446A |  | G3446A |  | G3446A |  |
| **Bangladesh clade_Specific** | P | C3452T | N349T |  |  |  |  | C3452T | N349T | C3452T | N349T | C3452T | N349T |  |  |
|  | P | C3456T | L351F |  |  |  |  | C3456T | L351F | C3456T | L351F | C3456T | L351F | C3456T | L351F |
|  | P | T3465A | C354S |  |  |  |  | T3465A | C354S | T3465A | C354S | T3465A | C354S | T3465A | C354S |
|  | P | G3470A |  |  |  | G3470A |  | G3470A |  | G3470A |  | G3470A |  | G3470A |  |
| **Bangladesh clade_Specific** | P | T3485C |  |  |  |  |  | T3485C |  | T3485C |  | T3485C |  |  |  |
|  | P | C3493T | P363L |  |  |  |  | C3493T | P363L | C3493T | P363L | C3493T | P363L | C3493T | P363L |
|  | P | C3498T | H365Y |  |  |  |  | C3498T | H365Y | C3498T | H365Y | C3498T | H365Y | C3498T | H365Y |
|  | P | T3501A | W366R |  |  |  |  | T3501A | W366R | T3501A | W366R | T3501A | W366R | T3501A | W366R |
| **Bangladesh clade_Specific** | P | A3504G | S367G |  |  |  |  | A3504G | S367G | A3504G | S367G | A3504G | S367G |  |  |
|  | P | C3509T |  |  |  |  |  | C3509T |  | C3509T |  | C3509T |  | C3509T |  |
|  | P | A3513G | R370G |  |  |  |  | A3513G | R370G | A3513G | R370G | A3513G | R370G | A3513G | R370G |
|  | P | A3518G |  |  |  |  |  | A3518G |  | A3518G |  | A3518G |  | A3518G |  |
|  | P | T3520G | I372R |  |  |  |  | T3520G | I372R | T3520G | I372R | T3520G | I372R | T3520G | I372R |
|  | P | G3527A |  |  |  |  |  | G3527A |  | G3527A |  | G3527A |  | G3527A |  |
| **Bangladesh clade_Specific** | P | T3530C |  |  |  |  |  | T3530C |  | T3530C |  | T3530C |  |  |  |
|  | P | A3533G |  |  |  | A3533G |  | A3533G |  | A3533G |  | A3533G |  | A3533G |  |
|  | P | G3543A | V380T |  |  |  |  | G3543A | V380T | G3543A | V380T | G3543A | V380T | G3543A | V380T |
|  | P | T3544C |  |  |  | T3544C |  | T3544C |  | T3544C |  | T3544C |  | T3544C |  |
|  | P | A3547G | N381S |  |  |  |  | A3547G | N381S | A3547G | N381S | A3547G | N381S | A3547G | N381S |
|  | P | G3550A | G382D |  |  |  |  | G3550A | G382D | G3550A | G382D | G3550A | G382D | G3550A | G382D |
|  | P | G3557A | V384A |  |  |  |  | G3557A | V384A | G3557A | V384A | G3557A | V384A | G3557A | V384A |
| **India_Specific** | P | C3562A | T386N |  |  |  |  |  |  |  |  |  |  | C3562A | T386N |
| **Bangladesh clade_Specific** | P | G3567A | D388N |  |  |  |  | G3567A | D388N | G3567A | D388N | G3567A | D388N |  |  |
|  | P | G3571A | R389K |  |  |  |  | G3571A | R389K | G3571A | R389K | G3571A | R389K | G3571A | R389K |
|  | P | A3575G |  |  |  | A3575G |  |  |  | A3575G |  |  |  |  |  |
|  | P | A3578G |  |  |  |  |  | A3578G |  | A3578G |  | A3578G |  | A3578G |  |
|  | P | G3599A |  |  |  |  |  | G3599A |  | G3599A |  | G3599A |  | G3599A |  |
|  | P | T3608C |  |  |  | T3608C |  | T3608C |  | T3608C |  | T3608C |  | T3608C |  |
|  | P | T3611C |  |  |  |  |  | T3611C |  | T3611C |  | T3611C |  | T3611C |  |
|  | P | C3614T |  |  |  | C3614T |  | C3614T |  | C3614T |  | C3614T |  | C3614T |  |
|  | P | C3634A | A410E |  |  |  |  | C3634A | A410E | C3634A | A410E | C3634A | A410E | C3634A | A410E |
| **Absent in 2023 India** | P | C3667T | P421L |  |  |  |  |  |  |  |  |  |  | C3667T | P421L |
| **2018 India_Specific** | P | **G3679A** | **S425N** |  |  |  |  |  |  |  |  |  |  | **G3679A** | **S425N** |
| **India_Specific** | P | A3751G | Q449R |  |  |  |  |  |  |  |  |  |  | A3751G | Q449R |
|  | P | C3753T |  |  |  |  |  | C3753T |  | C3753T |  | C3753T |  | C3753T |  |
|  | P | C3760T | A452V |  |  |  |  | C3760T | A452V | C3760T | A452V | C3760T | A452V | C3760T | A452V |
|  | P | T3762C | S453P |  |  |  |  | T3762C | S453P | T3762C | S453P | T3762C | S453P | T3762C | S453P |
|  | P | C3769T | A455V |  |  |  |  | C3769T | A455V | C3769T | A455V | C3769T | A455V | C3769T | A455V |
|  | P | T3773G |  |  |  | T3773G |  | T3773G |  | T3773G |  | T3773G |  | T3773G |  |
|  | P | A3778G | E458G |  |  |  |  |  |  |  |  |  |  | A3778G | E458G |
|  | P | G3788A |  |  |  |  |  | G3788A |  | G3788A |  | G3788A |  | G3788A |  |
|  | P | T3796C | V464A |  |  |  |  | T3796C | V464A | T3796C | V464A | T3796C | V464A | T3796C | V464A |
|  | P | C3803T |  |  |  |  |  | C3803T |  | C3803T |  | C3803T |  | C3803T |  |
|  | P | T3805C | V467A |  |  | T3805C | V467A | T3805C | V467A | T3805C | V467A | T3805C | V467A | T3805C | V467A |
|  | P | C3809T | D468G |  |  |  |  | C3809T | D468G | C3809T | D468G | C3809T | D468G | C3809T | D468G |
|  | P | C3836T |  |  |  |  |  | C3836T |  | C3836T |  | C3836T |  | C3836T |  |
|  | P | C3869T |  |  |  |  |  |  |  |  |  |  |  | C3869T |  |
|  | P | C3878T |  |  |  |  |  | C3878T |  | C3878T |  | C3878T |  | C3878T |  |
|  | P | G3893A |  |  |  |  |  | G3893A |  | G3893A |  | G3893A |  | G3893A |  |
|  | P | T3908C |  |  |  |  |  | T3908C |  | T3908C |  | T3908C |  | T3908C |  |
| **Bangladesh clade_Specific** | P | T3911C |  |  |  |  |  | T3911C |  | T3911C |  | T3911C |  |  |  |
|  | P | T3923C |  |  |  |  |  |  |  |  |  |  |  | T3923C |  |
|  | P | T3929C |  |  |  | T3929C |  | T3929C |  | T3929C |  | T3929C |  | T3929C |  |
|  | P | T3941C |  |  |  |  |  | T3941C |  | T3941C |  | T3941C |  | T3941C |  |
| **Bangladesh clade_Specific** | P | G3947A |  |  |  |  |  | G3947A |  | G3947A |  | G3947A |  |  |  |
|  | P | G3950A |  |  |  | G3950A |  | G3950A |  | G3950A |  | G3950A |  | G3950A |  |
|  | P | C3954T |  |  |  |  |  | C3954T |  | C3954T |  | C3954T |  | C3954T |  |
|  | P | A3956G |  |  |  | A3956G |  | A3956G |  | A3956G |  | A3956G |  | A3956G |  |
| **Bangladesh clade_Specific** | P | G3965A |  |  |  |  |  | G3965A |  | G3965A |  | G3965A |  |  |  |
|  | P | A3977C |  |  |  |  |  | A3977C |  | A3977C |  | A3977C |  | A3977C |  |
|  | P | T3995C |  |  |  | T3995C |  | T3995C |  | T3995C |  | T3995C |  | T3995C |  |
|  | P | T4002C |  |  |  |  |  | T4002C |  | T4002C |  | T4002C |  | T4002C |  |
|  | P | A4004G |  |  |  |  |  |  |  | A4004G |  | A4004G |  |  |  |
| **Bangladesh clade_Specific** | P | G4052A |  |  |  |  |  | G4052A |  | G4052A |  | G4052A |  |  |  |
|  | P | G4058A |  |  |  |  |  |  |  | G4058A |  |  |  | G4058A |  |
|  | P | C4061T |  |  |  |  |  | C4061T |  | C4061T |  | C4061T |  | C4061T |  |
| **Bangladesh clade_Specific** | P | A4070G |  |  |  |  |  | A4070G |  | A4070G |  | A4070G |  |  |  |
|  | P | G4082A |  |  |  |  |  | G4082A |  | G4082A |  | G4082A |  | G4082A |  |
|  | P | T4085C |  |  |  | T4085C |  | T4085C |  | T4085C |  | T4085C |  | T4085C |  |
|  | P | C4088T |  |  |  | C4088T |  | C4088T |  | C4088T |  | C4088T |  | C4088T |  |
|  | P | C4091T |  |  |  |  |  | C4091T |  | C4091T |  | C4091T |  | C4091T |  |
|  | P | C4103T |  |  |  |  |  | C4103T |  | C4103T |  | C4103T |  | C4103T |  |
|  | P | C4116T |  |  |  |  |  | C4116T |  | C4116T |  | C4116T |  | C4116T |  |
|  | P | A4142G |  |  |  |  |  | A4142G |  | A4142G |  | A4142G |  | A4142G |  |
|  | P | A4148G |  |  |  |  |  | A4148G |  | A4148G |  | A4148G |  | A4148G |  |
|  | P | G4151A |  |  |  |  |  |  |  | G4151A |  | G4151A |  |  |  |
|  | P | A4163G |  |  |  |  |  |  |  | A4163G |  | A4163G |  | A4163G |  |
|  | P | G4166A |  |  |  |  |  | G4166A |  | G4166A |  | G4166A |  | G4166A |  |
|  | P | G4169C |  |  |  | G4169C |  | G4169C |  | G4169C |  | G4169C |  | G4169C |  |
|  | P | A4174G | N590S |  |  | A4174G | N590S | A4174G | N590S | A4174G | N590S | A4174G | N590S | A4174G | N590S |
|  | P | C4208T |  |  |  |  |  | C4208T |  | C4208T |  | C4208T |  | C4208T |  |
|  | P | A4209G | I602V |  |  |  |  | A4209G | I602V | A4209G | I602V | A4209G | I602V | A4209G | I602V |
|  | P | A4214G |  |  |  |  |  | A4214G |  | A4214G |  | A4214G |  | A4214G |  |
|  | P | G4220A |  |  |  |  |  |  |  | G4220A |  |  |  |  |  |
|  | P | T4226C |  |  |  |  |  |  |  |  |  |  |  | T4226C |  |
| **2018 India & Bangladesh Specific** | P | T4229A |  |  |  |  |  |  |  | T4229A |  | T4229A |  | T4229A |  |
|  | P | T4232C |  |  |  | T4232C |  | T4232C |  | T4232C |  | T4232C |  | T4232C |  |
|  | P | T4235A |  |  |  |  |  | T4235A |  | T4235A |  | T4235A |  | T4235A |  |
|  | P | T4262C |  |  |  |  |  |  |  |  |  |  |  | T4262C |  |
|  | P | A4265G |  |  |  |  |  | A4265G |  | A4265G |  | A4265G |  | A4265G |  |
|  | P | C4277T |  |  |  |  |  | C4277T |  | C4277T |  | C4277T |  | C4277T |  |
| **India_2023+Bangladesh Specific** | P | G4283A |  |  |  |  |  |  |  | G4283A |  | G4283A |  | G4283A |  |
| **India_Specific** | P | G4290A | A629T |  |  |  |  |  |  |  |  |  |  | G4290A | A629T |
|  | P | A4298C |  |  |  |  |  | A4298C |  | A4298C |  | A4298C |  | A4298C |  |
|  | P | G4301A |  |  |  | G4301A |  | G4301A |  | G4301A |  | G4301A |  | G4301A |  |
|  | P | A4309G | E635G |  |  |  |  | A4309G | E635G | A4309G | E635G | A4309G | E635G | A4309G | E635G |
|  | P | T4316G |  |  |  |  |  | T4316G |  | T4316G |  | T4316G |  | T4316G |  |
|  | P | T4322C |  |  |  |  |  |  |  | T4322C |  |  |  |  |  |
|  | P | T4331C |  |  |  |  |  | T4331C |  | T4331C |  | T4331C |  | T4331C |  |
|  | P | T4334C |  |  |  |  |  |  |  | T4334C |  | T4334C |  | T4334C |  |
|  | P | T4337C |  |  |  |  |  |  |  | T4337C |  |  |  |  |  |
|  | P | G4343A |  |  |  |  |  | G4343A |  | G4343A |  | G4343A |  | G4343A |  |
|  | P | A4346G |  |  |  |  |  | A4346G |  | A4346G |  | A4346G |  | A4346G |  |
|  | P | A4352G |  |  |  |  |  | A4352G |  | A4352G |  | A4352G |  | A4352G |  |
|  | P | A4358G |  |  |  |  |  | A4358G |  | A4358G |  | A4358G |  | A4358G |  |
|  | P | T4367C |  |  |  |  |  | T4367C |  | T4367C |  | T4367C |  | T4367C |  |
|  | P | A4368G | M655V |  |  |  |  |  |  | A4368G | M655V | A4368G | M655V |  |  |
|  | P | A4382G |  |  |  |  |  | A4382G |  | A4382G |  | A4382G |  | A4382G |  |
|  | P | T4391C |  |  |  |  |  | T4391C |  | T4391C |  | T4391C |  | T4391C |  |
|  | P | A4395G | I664V |  |  | A4395G | I664V | A4395G | I664V | A4395G | I664V | A4395G | I664V | A4395G | I664V |
|  | P | C4418T |  |  |  |  |  | C4418T |  | C4418T |  | C4418T |  | C4418T |  |
|  | P | A4424G |  |  |  |  |  | A4424G |  | A4424G |  | A4424G |  | A4424G |  |
|  | P | G4433A | E676K |  |  |  |  | G4433A | E676K | G4433A | E676K | G4433A | E676K | G4433A | E676K |
| **India_Specific** | P | G4453A | G683D |  |  |  |  |  |  |  |  |  |  | G4453A | G683D |
|  | P | C4457T |  |  |  |  |  |  |  | C4457T |  | C4457T |  |  |  |
|  | P | A4465G | K687R |  |  |  |  | A4465G | K687R | A4465G | K687R | A4465G | K687R | A4465G | K687R |
|  | P | G4469A |  |  |  |  |  | G4469A |  | G4469A |  | G4469A |  | G4469A |  |
|  | P | G4499A |  |  |  | G4499A |  | G4499A |  | G4499A |  | G4499A |  | G4499A |  |
|  | P | C4502T |  |  |  |  |  | C4502T |  | C4502T |  | C4502T |  | C4502T |  |
|  | P | C4517T |  |  |  |  |  |  |  | C4517T |  | C4517T |  |  |  |
| **Present in all clades** | P | T4529C |  | T4529C |  | T4529C |  | T4529C |  | T4529C |  | T4529C |  | T4529C |  |
|  | V | C3635A |  |  |  |  |  | C3635A |  | C3635A |  | C3635A |  | C3635A |  |
| **Absent in 2023 India** | V | C3668T |  |  |  |  |  |  |  |  |  |  |  | C3668T |  |
| **2018 India_Specific** | V | **G3680A** |  |  |  |  |  |  |  |  |  |  |  | **G3680A** |  |
| **India_Specific** | V | A3752G |  |  |  |  |  |  |  |  |  |  |  | A3752G |  |
|  | V | C3754T | T450I |  |  |  |  | C3754T | T450I | C3754T | T450I | C3754T | T450I | C3754T | T450I |
|  | V | C3761T | C452R |  |  |  |  | C3761T | C452R | C3761T | C452R | C3761T | C452R | C3761T | C452R |
|  | V | T3763C | F453S |  |  |  |  | T3763C | F453S | T3763C | F453S | T3763C | F453S | T3763C | F453S |
|  | V | C3770T |  |  |  |  |  | C3770T |  | C3770T |  | C3770T |  | C3770T |  |
|  | V | G3771T | G456C |  |  |  |  | G3771T | G456C | G3771T | G456C | G3771T | G456C | G3771T | G456C |
|  | V | T3774G | *457E |  |  | T3774G | *457E | T3774G | *457E | T3774G | *457E | T3774G | *457E | T3774G | *457E |
|  | W | C3633A |  |  |  |  |  | C3633A |  | C3633A |  | C3633A |  | C3633A |  |
| **Absent in 2023 India** | W | C3666T |  |  |  |  |  |  |  |  |  |  |  | C3666T |  |
| **2018 India_Specific** | W | **G3678A** | **V425M** |  |  |  |  |  |  |  |  |  |  | **G3678A** | **V425M** |
| **India_Specific** | W | A3750G | N449D |  |  |  |  |  |  |  |  |  |  | A3750G | N449D |
|  | C | T2501C | V25A |  |  | T2501C | V25A | T2501C | V25A | T2501C | T2501C | T2501C | T2501C | T2501C | V25A |
|  | C | T2525C | I33T |  |  | T2525C | I33T | T2525C | I33T | T2525C | T2525C | T2525C | T2525C | T2525C | I33T |
|  | C | A2527G | K34E |  |  | A2527G | K34E | A2527G | K34E | A2527G | Q41R | A2527G | Q41R | A2527G | K34E |
| **India_Specific** | C | T2546C | I40T |  |  |  |  |  |  |  |  |  |  | T2546C | I40T |
|  | C | C2550T |  |  |  |  |  |  |  |  |  |  |  | C2550T |  |
| **2023 India_Specific** | C | **G2616A** |  |  |  |  |  |  |  |  |  |  |  | **G2616A** |  |
|  | C | A2646G |  |  |  |  |  |  |  | A2646G |  | A2646G |  |  |  |
| **2023 India_Specific** | C | **T2649C** |  |  |  |  |  |  |  |  |  |  |  | **T2649C** |  |
|  | C | T2820C |  |  |  |  |  | T2820C |  | T2820C | Y139H | T2820C | Y139H | T2820C |  |
|  | C | A2823T |  |  |  | A2823T |  | A2823T |  | A2823T | T140S | A2823T | T140S | A2823T |  |
|  | M | C5314T |  |  |  |  |  |  |  |  |  |  |  | C5314T |  |
|  | M | A5333C |  |  |  |  |  | A5333C |  | A5333C |  | A5333C |  | A5333C |  |
|  | M | A5353G |  |  |  |  |  | A5353G |  | A5353G |  | A5353G |  | A5353G |  |
|  | M | C5356T |  |  |  |  |  | C5356T |  | C5356T |  | C5356T |  | C5356T |  |
|  | M | G5362A |  |  |  |  |  |  |  | G5362A |  | G5362A |  | G5362A |  |
|  | M | C5383T |  |  |  |  |  |  |  |  |  |  |  | C5383T |  |
|  | M | C5443T |  |  |  |  |  |  |  | C5443T |  | C5443T |  | C5443T |  |
|  | M | A5461G |  |  |  | A5461G |  | A5461G |  | A5461G |  | A5461G |  | A5461G |  |
| **Present in all clades** | M | A5546G | S147G | A5546G | S147G | A5546G | S147G | A5546G | S147G | A5546G | S147G | A5546G | S147G | A5546G | S147G |
| **Bangladesh clade_Specific** | M | C5569T |  |  |  |  |  | C5569T |  | C5569T |  | C5569T |  |  |  |
|  | M | C5584T |  |  |  |  |  |  |  |  |  |  |  | C5584T |  |
| **Present in all clades** | M | T5602C |  | T5602C |  | T5602C |  | T5602C |  | T5602C |  | T5602C |  | T5602C |  |
| **Bangladesh clade_Specific** | M | G5647A |  |  |  |  |  | G5647A |  | G5647A |  | G5647A |  |  |  |
|  | M | A5701G |  |  |  |  |  |  |  |  |  |  |  | A5701G |  |
|  | M | A5728G |  |  |  |  |  | A5728G |  | A5728G |  | A5728G |  | A5728G |  |
|  | M | T5735C |  |  |  |  |  | T5735C |  | T5735C |  | T5735C |  | T5735C |  |
|  | M | T5753C |  |  |  |  |  | T5753C |  | T5753C |  | T5753C |  | T5753C |  |
| **Bangladesh clade_Specific** | M | G5767A |  |  |  |  |  | G5767A |  | G5767A |  | G5767A |  |  |  |
|  | M | T5785C |  |  |  |  |  | T5785C |  | T5785C |  | T5785C |  | T5785C |  |
|  | M | G5800A |  |  |  |  |  | G5800A |  | G5800A |  | G5800A |  | G5800A |  |
|  | M | C5806T |  |  |  |  |  | C5806T |  | C5806T |  | C5806T |  | C5806T |  |
|  | M | A5818G |  |  |  |  |  | A5818G |  | A5818G |  | A5818G |  | A5818G |  |
|  | M | G5827A |  |  |  |  |  | G5827A |  | G5827A |  | G5827A |  | G5827A |  |
|  | M | T5842C |  |  |  |  |  | T5842C |  | T5842C |  | T5842C |  | T5842C |  |
|  | M | G5848A |  |  |  |  |  | G5848A |  | G5848A |  | G5848A |  | G5848A |  |
|  | M | T5854C |  |  |  |  |  | T5854C |  | T5854C |  | T5854C |  | T5854C |  |
|  | M | C5857T |  |  |  |  |  | C5857T |  | C5857T |  | C5857T |  | C5857T |  |
|  | M | G5878A |  |  |  | G5878A |  | G5878A |  | G5878A |  | G5878A |  | G5878A |  |
| **2023 India_Specific** | M | **T5887C** |  |  |  |  |  |  |  |  |  |  |  | **T5887C** |  |
|  | M | C5959T |  |  |  |  |  |  |  |  |  |  |  | C5959T |  |
|  | M | A5986T |  |  |  |  |  | A5986T |  | A5986T |  | A5986T |  | A5986T |  |
|  | M | C6007T |  |  |  |  |  | C6007T |  | C6007T |  | C6007T |  | C6007T |  |
|  | M | T6010C |  |  |  | T6010C |  | T6010C |  | T6010C |  | T6010C |  | T6010C |  |
|  | M | C6019T |  |  |  |  |  | C6019T |  | C6019T |  | C6019T |  | C6019T |  |
|  | M | C6022T |  |  |  |  |  | C6022T |  | C6022T |  | C6022T |  | C6022T |  |
| **2023 India_Specific** | M | **C6034T** |  |  |  |  |  |  |  |  |  |  |  | **C6034T** |  |
|  | M | A6098G | I331V |  |  | A6098G | I331V | A6098G | I331V | A6098G | I331V | A6098G | I331V | A6098G | I331V |
| **2018 India_Specific** | F | **C6163T** |  |  |  |  |  |  |  |  |  |  |  | **C6163T** |  |
|  | F | G6691C | C11S |  |  | G6691C | C11S | G6691C | C11S | G6691C | C11S | G6691C | C11S | G6691C | C11S |
| **India_Specific** | F | A6702T | I15L |  |  |  |  |  |  |  |  |  |  | A6702T | I15L |
|  | F | **T6705C** |  |  |  | T6705C |  | T6705C |  | T6705C |  | T6705C |  | T6705C |  |
| **2023 India_Specific** | F | **G6722A** |  |  |  |  |  |  |  |  |  |  |  | **G6722A** |  |
|  | F | G6783A | V42I |  |  | G6783A | V42I | G6783A | V42I | G6783A | V42I | G6783A | V42I | G6783A | V42I |
| **Bangladesh clade_Specific** | F | A6875G |  |  |  |  |  | **A6875G** |  | **A6875G** |  | **A6875G** |  |  |  |
|  | F | **T6893C** |  |  |  |  |  |  |  | **T6893C** |  |  |  |  |  |
| **India_Specific** | F | C6917T |  |  |  |  |  |  |  |  |  |  |  | C6917T |  |
|  | F | **T6918C** |  |  |  |  |  | T6918C |  | T6918C |  | T6918C |  | T6918C |  |
| **Bangladesh clade_Specific** | F | A6923G | **T88M** |  |  |  |  | **A6923G** | **T88M** | **A6923G** | **T88M** | **A6923G** | **T88M** |  |  |
| **Bangladesh clade_Specific** | F | A6953G |  |  |  |  |  | **A6953G** |  | **A6953G** |  | **A6953G** |  |  |  |
| **2023 India_Specific** | F | **C6956T** |  |  |  |  |  |  |  |  |  |  |  | **C6956T** |  |
| **2019_India_specific** | F | C6968T |  |  |  |  |  |  |  |  |  |  |  | C6968T |  |
|  | F | G6983A | V108L |  |  |  |  | G6983A | V108L | G6983A | V108L | G6983A | V108L | G6983A | V108L |
| **Bangladesh clade_Specific** | F | T6987C |  |  |  |  |  | **T6987C** |  | **T6987C** |  | **T6987C** |  |  |  |
|  | F | A6989G |  |  |  |  |  | A6989G |  | A6989G |  | A6989G |  | A6989G |  |
|  | F | C7031T |  |  |  |  |  |  |  | C7031T |  | C7031T |  | C7031T |  |
|  | F | C7043T |  |  |  |  |  | C7043T |  | C7043T |  | C7043T |  | C7043T |  |
|  | F | C7059T |  |  |  | C7059T |  | C7059T |  | C7059T |  | C7059T |  | C7059T |  |
|  | F | G7076A |  |  |  | G7076A |  | G7076A |  | G7076A |  | G7076A |  | G7076A |  |
|  | F | T7112A |  |  |  |  |  | T7112A |  | T7112A |  | T7112A |  | T7112A |  |
|  | F | T7178C |  |  |  |  |  | T7178C |  | T7178C |  |  |  | T7178C |  |
|  | F | C7182T |  | C7182T |  |  |  | C7182T |  | C7182T |  | C7182T |  | C7182T |  |
|  | F | T7205C |  |  |  | T7205C |  | T7205C |  | T7205C |  | T7205C |  | T7205C |  |
|  | F | T7206C |  |  |  |  |  |  |  |  |  |  |  | T7206C |  |
|  | F | A7208G |  |  |  | A7208G |  | A7208G |  | A7208G |  | A7208G |  | A7208G |  |
|  | F | C7254T |  |  |  |  |  |  |  | C7254T |  | C7254T |  | C7254T |  |
|  | F | G7262A |  |  |  |  |  | G7262A |  | G7262A |  | G7262A |  | G7262A |  |
|  | F | T7266C |  |  |  |  |  | T7266C |  | T7266C |  | T7266C |  | T7266C |  |
|  | F | C7304T |  |  |  |  |  | C7304T |  | C7304T |  | C7304T |  | C7304T |  |
|  | F | T7328C |  |  |  |  |  |  |  |  |  |  |  | T7328C |  |
|  | F | **T7349A** |  |  |  |  |  |  |  |  |  |  |  | T7349A |  |
|  | F | **A7394G** |  |  |  |  |  | A7394G |  | A7394G |  | A7394G |  | A7394G |  |
|  | F | **A7448G** |  |  |  | A7448G |  |  |  | A7448G |  | A7448G |  | A7448G |  |
|  | F | **A7727G** |  |  |  | A7727G |  | A7727G |  | A7727G |  | A7727G |  | A7727G |  |
|  | F | **A7733G** |  |  |  |  |  | A7733G |  | A7733G |  | A7733G |  | A7733G |  |
|  | F | **C7781A** |  |  |  |  |  |  |  |  |  |  |  | C7781A |  |
| **Bangladesh clade_Specific** | F | T7826C |  |  |  |  |  | T7826C |  | T7826C |  | T7826C |  |  |  |
|  | F | **T7829C** |  |  |  | T7829C |  | T7829C |  | T7829C |  | T7829C |  | T7829C |  |
|  | F | **T7835C** |  |  |  | T7835C |  | T7835C |  | T7835C |  | T7835C |  | T7835C |  |
|  | F | **A7868G** |  |  |  | A7868G |  | A7868G |  | A7868G |  | A7868G |  | A7868G |  |
|  | F | **C7884T** |  |  |  |  |  |  |  |  |  |  |  | C7884T |  |
|  | F | **C7919T** |  |  |  |  |  | C7919T |  | C7919T |  | C7919T |  | C7919T |  |
| **Bangladesh clade_Specific** | F | A7946G |  |  |  |  |  | A7946G |  | A7946G |  | A7946G |  |  |  |
|  | F | **T8069C** |  |  |  |  |  |  |  |  |  |  |  | T8069C |  |
|  | F | **T8078C** |  |  |  |  |  |  |  |  |  |  |  | T8078C |  |
| **Bangladesh clade_Specific** | F | G8087A |  |  |  |  |  | G8087A |  | G8087A |  | G8087A |  |  |  |
|  | F | **T8157C** |  |  |  | T8157C |  | T8157C |  | T8157C |  | T8157C |  | T8157C |  |
|  | F | **G8174A** |  |  |  |  |  |  |  |  |  |  |  | G8174A |  |
|  | F | **G8183A** |  |  |  |  |  | G8183A |  | G8183A |  |  |  | G8183A |  |
|  | F | **T8189C** |  |  |  |  |  |  |  |  |  |  |  | T8189C |  |
|  | F | **A8219G** |  |  |  |  |  |  |  |  |  |  |  | A8219G |  |
|  | F | **C8234T** |  |  |  |  |  | C8234T |  | C8234T |  | C8234T |  | C8234T |  |
|  | F | **C8270T** |  |  |  |  |  |  |  | C8270T |  |  |  |  |  |
|  | F | **C8285T** |  |  |  | C8285T |  | C8285T |  | C8285T |  | C8285T |  | C8285T |  |
| **Bangladesh clade_Specific** | F | A8297T |  |  |  |  |  | A8297T |  | A8297T |  | A8297T |  |  |  |
|  | G | G8955A | A3T | G8955A | A3T |  |  | G8955A | A3T | G8955A | A3T | G8955A | A3T | G8955A | A3T |
|  | G | A8962G | N5S |  |  | A8962G | N5S | A8962G | N5S | A8962G | N5S | A8962G | N5S | A8962G | N5S |
|  | G | A8988G | T14A |  |  |  |  | A8988G | T14A | A8988G | T14A | A8988G | T14A | A8988G | T14A |
| **Bangladesh clade_Specific** | G | A8999G |  |  |  |  |  | A8999G |  | A8999G |  | A8999G |  |  |  |
|  | G | T9007A | I20N | T9007A | I20N |  |  | T9007A | I20N | T9007A | I20N | T9007A | I20N | T9007A | I20N |
|  | G | **C9035T** |  |  |  |  |  |  |  | C9035T |  |  |  |  |  |
|  | G | A9056G |  |  |  |  |  |  |  |  |  |  |  | A9056G |  |
|  | G | A9068G |  |  |  |  |  | A9068G |  | A9068G |  | A9068G |  | A9068G |  |
|  | G | A9083G |  |  |  |  |  | A9083G |  | A9083G |  | A9083G |  | A9083G |  |
|  | G | A9107G |  |  |  |  |  | A9107G |  | A9107G |  | A9107G |  | A9107G |  |
|  | G | T9114C |  |  |  |  |  | T9114C |  | T9114C |  | T9114C |  | T9114C |  |
|  | G | T9125C |  |  |  |  |  | T9125C |  | T9125C |  | T9125C |  | T9125C |  |
|  | G | C9128T |  |  |  |  |  | C9128T |  | C9128T |  | C9128T |  | C9128T |  |
|  | G | G9131A |  |  |  |  |  | G9131A |  | G9131A |  | G9131A |  | G9131A |  |
|  | G | A9161G |  |  |  |  |  | A9161G |  |  |  | A9161G |  | A9161G |  |
|  | G | T9164C |  |  |  |  |  | T9164C |  | T9164C |  | T9164C |  | T9164C |  |
|  | G | C9182T |  |  |  |  |  | C9182T |  | C9182T |  | C9182T |  | C9182T |  |
|  | G | G9192A | V82M |  |  |  |  | G9192A | V82M | G9192A | V82M | G9192A | V82M | G9192A | V82M |
|  | G | **A9200G** |  |  |  |  |  |  |  |  |  |  |  | A9200G |  |
|  | G | G9206A |  |  |  |  |  | G9206A |  | G9206A |  | G9206A |  | G9206A |  |
|  | G | G9213A | G89S |  |  |  |  | G9213A | G89S | G9213A | G89S | G9213A | G89S | G9213A | G89S |
|  | G | A9221G |  |  |  |  |  | A9221G |  | A9221G |  | A9221G |  | A9221G |  |
| **Bangladesh clade_Specific** | G | A9233G |  |  |  |  |  | A9233G |  | A9233G |  | A9233G |  |  |  |
|  | G | T9242C |  |  |  |  |  | T9242C |  | T9242C |  | T9242C |  | T9242C |  |
|  | G | C9251T |  |  |  |  |  | C9251T |  | C9251T |  | C9251T |  | C9251T |  |
|  | G | A9257G |  |  |  |  |  |  |  |  |  |  |  | A9257G |  |
|  | G | C9269G |  |  |  |  |  | C9269G |  | C9269G |  | C9269G |  | C9269G |  |
| **2018 India & Bangladesh Specific** | G | C9287T |  |  |  |  |  | C9287T |  | C9287T |  | C9287T |  | C9287T |  |
|  | G | C9299T |  |  |  | C9299T |  | C9299T |  | C9299T |  | C9299T |  | C9299T |  |
|  | G | T9302C |  |  |  |  |  | T9302C |  | T9302C |  | T9302C |  | T9302C |  |
|  | G | C9317T |  |  |  |  |  | C9317T |  | C9317T |  | C9317T |  | C9317T |  |
|  | G | C9344T |  |  |  |  |  |  |  |  |  |  |  | C9344T |  |
|  | G | G9350A |  |  |  |  |  | G9350A |  | G9350A |  | G9350A |  | G9350A |  |
| **2019_India_specific** | G | A9351G | T135A |  |  |  |  |  |  |  |  |  |  | A9351G | T135A |
|  | G | C9392T |  |  |  |  |  | C9392T |  | C9392T |  | C9392T |  | C9392T |  |
|  | G | C9404T |  |  |  |  |  |  |  |  |  |  |  | C9404T |  |
|  | G | G9463A | R172K |  |  |  |  | G9463A | R172K | G9463A | R172K | G9463A | R172K | G9463A | R172K |
|  | G | A9467G |  |  |  | A9467G |  | A9467G |  | A9467G |  | A9467G |  | A9467G |  |
|  | G | G9479A |  |  |  | G9479A |  | G9479A |  | G9479A |  | G9479A |  | G9479A |  |
| **Present in all clades** | G | A9491G |  | A9491G |  | A9491G |  | A9491G |  | A9491G |  | A9491G |  | A9491G |  |
|  | G | T9512C |  |  |  |  |  | T9512C |  | T9512C |  | T9512C |  | T9512C |  |
|  | G | C9515T |  |  |  |  |  | C9515T |  | C9515T |  | C9515T |  | C9515T |  |
|  | G | T9540C |  |  |  | T9540C |  | T9540C |  | T9540C |  | T9540C |  | T9540C |  |
|  | G | G9545A |  |  |  |  |  | G9545A |  | G9545A |  | G9545A |  | G9545A |  |
|  | G | T9557C |  |  |  |  |  |  |  |  |  |  |  | T9557C |  |
|  | G | T9566C |  |  |  |  |  | T9566C |  | T9566C |  | T9566C |  | T9566C |  |
|  | G | T9581C |  |  |  |  |  |  |  |  |  |  |  | T9581C |  |
|  | G | **A9584G** |  |  |  |  |  |  |  |  |  |  |  | A9584G |  |
|  | G | T9590C |  |  |  |  |  | T9590C |  | T9590C |  | T9590C |  | T9590C |  |
| **Bangladesh clade_Specific** | G | T9609C |  |  |  |  |  | T9609C |  | T9609C |  | T9609C |  |  |  |
|  | G | C9623T |  |  |  |  |  | C9623T |  | C9623T |  | C9623T |  | C9623T |  |
|  | G | **T9630C** | Y228H |  |  |  |  |  |  | T9630C | Y228H | T9630C | Y228H |  |  |
|  | G | T9632C |  |  |  |  |  | T9632C |  | T9632C |  | T9632C |  | T9632C |  |
|  | G | G9655A | R236K |  |  |  |  | G9655A | R236K | G9655A | R236K | G9655A | R236K | G9655A | R236K |
|  | G | T9728C |  |  |  |  |  | T9728C |  | T9728C |  | T9728C |  | T9728C |  |
|  | G | T9734A |  |  |  |  |  | T9734A |  | T9734A |  | T9734A |  | T9734A |  |
|  | G | A9743G |  |  |  |  |  | A9743G |  | A9743G |  | A9743G |  | A9743G |  |
|  | G | T9746C |  |  |  |  |  |  |  |  |  |  |  | T9746C |  |
|  | G | C9752T |  |  |  |  |  | C9752T |  | C9752T |  | C9752T |  | C9752T |  |
|  | G | T9755C |  |  |  |  |  | T9755C |  | T9755C |  | T9755C |  | T9755C |  |
|  | G | C9768T | P274S |  |  |  |  | C9768T | P274S | C9768T | P274S | C9768T | P274S | C9768T | P274S |
|  | G | C9791T |  |  |  |  |  | C9791T |  | C9791T |  | C9791T |  | C9791T |  |
|  | G | T9794C |  |  |  |  |  | T9794C |  | T9794C |  | T9794C |  | T9794C |  |
|  | G | T9800C |  |  |  |  |  | T9800C |  | T9800C |  | T9800C |  | T9800C |  |
|  | G | A9803G |  |  |  |  |  | A9803G |  | A9803G |  | A9803G |  | A9803G |  |
| **India_Specific** | G | A9811G | N288S |  |  |  |  |  |  |  |  |  |  | A9811G | N288S |
|  | G | A9827G |  |  |  | A9827G |  | A9827G |  | A9827G |  | A9827G |  | A9827G |  |
|  | G | A9843G | T299V |  |  |  |  | A9843G | T299V | A9843G | T299V | A9843G | T299V | A9843G | T299V |
|  | G | C9844T |  |  |  |  |  | C9844T |  | C9844T |  | C9844T |  | C9844T |  |
| **2018 India_Specific** | G | **C9872T** |  |  |  |  |  |  |  |  |  |  |  | **C9872T** |  |
|  | G | A9884G |  |  |  |  |  |  |  |  |  |  |  | A9884G |  |
|  | G | T9887A |  |  |  |  |  | T9887A |  | T9887A |  | T9887A |  | T9887A |  |
|  | G | C9899T |  |  |  |  |  | C9899T |  | C9899T |  | C9899T |  | C9899T |  |
|  | G | G9911A |  |  |  | G9911A |  | G9911A |  | G9911A |  | G9911A |  | G9911A |  |
|  | G | C9917T |  |  |  |  |  | C9917T |  | C9917T |  | C9917T |  | C9917T |  |
|  | G | G9922A | S325N |  |  |  |  | G9922A | S325N | G9922A | S325N | G9922A | S325N | G9922A | S325N |
|  | G | T9926C |  |  |  |  |  |  |  |  |  |  |  | T9926C |  |
|  | G | G9931A | G328E |  |  |  |  | G9931A | G328E | G9931A | G328E | G9931A | G328E | G9931A | G328E |
|  | G | G9933A | G329S |  |  |  |  | G9933A | G329S | G9933A | G329S | G9933A | G329S | G9933A | G329S |
|  | G | C9951T | L335F |  |  |  |  | C9951T | L335F | C9951T | L335F | C9951T | L335F | C9951T | L335F |
|  | G | C9957T | L337F |  |  |  |  | C9957T | L337F | C9957T | L337F | C9957T | L337F | C9957T | L337F |
|  | G | A9962G |  |  |  |  |  | A9962G |  | A9962G |  | A9962G |  | A9962G |  |
|  | G | G9964A | S339N |  |  |  |  | G9964A | S339N | G9964A | S339N | G9964A | S339N | G9964A | S339N |
|  | G | C9968T |  |  |  |  |  | C9968T |  | C9968T |  | C9968T |  | C9968T |  |
|  | G | G9979T | R344K |  |  |  |  | G9979T | R344K | G9979T | R344K | G9979T | R344K | G9979T | R344K |
|  | G | G9998A |  |  |  |  |  | G9998A |  | G9998A |  | G9998A |  | G9998A |  |
|  | G | T10007C |  |  |  |  |  | T10007C |  | T10007C |  | T10007C |  | T10007C |  |
|  | G | G10022A |  |  |  |  |  | G10022A |  | G10022A |  | G10022A |  | G10022A |  |
|  | G | T10037C |  |  |  |  |  | T10037C |  | T10037C |  | T10037C |  | T10037C |  |
|  | G | T10073C |  |  |  | T10073C |  | T10073C |  | T10073C |  | T10073C |  | T10073C |  |
|  | G | **A10075C** | K376T |  |  |  |  |  |  | A10075C | K376T |  |  |  |  |
| **India_Specific** | G | A10098G | I384V |  |  |  |  |  |  |  |  |  |  | A10098G | I384V |
|  | G | A10101G | T385A |  |  |  |  | A10101G | T385A | A10101G | T385A | A10101G | T385A | A10101G | T385A |
| **Bangladesh clade_Specific** | G | A10104G | K386E |  |  |  |  | A10104G | K386E | A10104G | K386E | A10104G | K386E |  |  |
|  | G | T10118C |  |  |  |  |  | T10118C |  | T10118C |  | T10118C |  | T10118C |  |
|  | G | T10130C |  |  |  |  |  | T10130C |  | T10130C |  | T10130C |  | T10130C |  |
|  | G | **T10151C** |  |  |  |  |  |  |  | T10151C |  | T10151C |  |  |  |
|  | G | C10163T |  |  |  |  |  | C10163T |  | C10163T |  | C10163T |  | C10163T |  |
|  | G | C10172T | I408V |  |  |  |  |  |  |  |  |  |  | C10172T | I408V |
|  | G | **T10181C** |  |  |  |  |  |  |  | T10181C |  |  |  |  |  |
|  | G | T10188C |  |  |  |  |  | T10188C |  | T10188C |  | T10188C |  | T10188C |  |
| **Bangladesh clade_Specific** | G | A10205G |  |  |  |  |  | A10205G |  | A10205G |  | A10205G |  |  |  |
|  | G | G10210A | G421E |  |  |  |  | G10210A | G421E | G10210A | G421E | G10210A | G421E | G10210A | G421E |
| **2019_India_specific** | G | A10215G | N423D |  |  |  |  |  |  |  |  |  |  | A10215G | N423D |
|  | G | C10218T | P424S |  |  |  |  | C10218T | P424S | C10218T | P424S | C10218T | P424S | C10218T | P424S |
|  | G | C10220T |  |  |  | C10220T |  | C10220T |  | C10220T |  | C10220T |  | C10220T |  |
|  | G | G10224A | V426I |  |  | G10224A | V426I | G10224A | V426I | G10224A | V426I | G10224A | V426I | G10224A | V426I |
| **India_Specific** | G | G10227A | V427I |  |  |  |  |  |  |  |  |  |  | G10227A | V427I |
|  | G | T10254C | L436Q |  |  |  |  | T10254C | L436Q | T10254C | L436Q | T10254C | L436Q | T10254C | L436Q |
| **2018 India_Specific** | G | **T10289C** |  |  |  |  |  |  |  |  |  |  |  | **T10289C** |  |
|  | G | **G10316A** |  |  |  |  |  |  |  | G10316A |  |  |  |  |  |
|  | G | A10319T |  |  |  |  |  | A10319T |  | A10319T |  | A10319T |  | A10319T |  |
|  | G | T10331C |  |  |  |  |  | T10331C |  | T10331C |  | T10331C |  | T10331C |  |
|  | G | **T10355C** |  |  |  |  |  | T10355C |  | T10355C |  | T10355C |  | T10355C |  |
|  | G | T10357A | L470Q |  |  | T10357A | L470Q | T10357A | L470Q | T10357A | L470Q | T10357A | L470Q | T10357A | L470Q |
|  | G | C10364T |  |  |  |  |  | C10364T |  | C10364T |  | C10364T |  | C10364T |  |
|  | G | C10371T |  |  |  | C10371T |  | C10371T |  | C10371T |  | C10371T |  | C10371T |  |
| **Bangladesh clade_Specific** | G | G10373A |  |  |  |  |  | G10373A |  | G10373A |  | G10373A |  |  |  |
|  | G | T10376C |  |  |  |  |  |  |  |  |  |  |  | T10376C |  |
|  | G | C10379A |  |  |  | C10379A |  | C10379A |  | C10379A |  | C10379A |  | C10379A |  |
|  | G | A10389G | N481D |  |  | A10389G | N481D | A10389G | N481D | A10389G | N481D | A10389G | N481D | A10389G | N481D |
|  | G | T10391C |  |  |  |  |  | T10391C |  | T10391C |  | T10391C |  | T10391C |  |
|  | G | A10403C |  |  |  |  |  | A10403C |  | A10403C |  | A10403C |  | A10403C |  |
|  | G | C10412T |  |  |  | C10412T |  | C10412T |  | C10412T |  | C10412T |  | C10412T |  |
|  | G | T10439C |  |  |  |  |  | T10439C |  | T10439C |  | T10439C |  | T10439C |  |
| **Bangladesh clade_Specific** | G | C10441A | T498K |  |  |  |  | C10441A | T498K | C10441A | T498K | C10441A | T498K |  |  |
|  | G | A10442G |  |  |  |  |  | A10442G |  | A10442G |  | A10442G |  | A10442G |  |
|  | G | T10445C |  |  |  |  |  | T10445C |  | T10445C |  | T10445C |  | T10445C |  |
|  | G | A10452G | I502V |  |  |  |  | A10452G | I502V | A10452G | I502V | A10452G | I502V | A10452G | I502V |
|  | G | A10466G |  |  |  |  |  | A10466G |  | A10466G |  | A10466G |  | A10466G |  |
|  | G | A10481C |  |  |  |  |  | A10481C |  | A10481C |  | A10481C |  | A10481C |  |
|  | G | A10487G |  |  |  |  |  | A10487G |  | A10487G |  | A10487G |  | A10487G |  |
| **Bangladesh clade_Specific** | G | C10493T |  |  |  |  |  | C10493T |  | C10493T |  | C10493T |  |  |  |
|  | G | C10511T |  |  |  |  |  | C10511T |  |  |  | C10511T |  | C10511T |  |
|  | G | T10535C |  |  |  |  |  | T10535C |  | T10535C |  | T10535C |  | T10535C |  |
|  | G | A10547G |  |  |  |  |  | A10547G |  | A10547G |  | A10547G |  | A10547G |  |
|  | G | A10581G | I545V |  |  |  |  | A10581G | I545V | A10581G | I545V | A10581G | I545V | A10581G | I545V |
|  | G | T10589C |  |  |  |  |  | T10589C |  | T10589C |  | T10589C |  | T10589C |  |
|  | G | G10592A |  |  |  | G10592A |  | G10592A |  | G10592A |  | G10592A |  | G10592A |  |
|  | G | G10601A |  |  |  |  |  | G10601A |  | G10601A |  | G10601A |  | G10601A |  |
|  | G | T10604C |  |  |  |  |  | T10604C |  |  |  | T10604C |  | T10604C |  |
|  | G | T10607C |  |  |  |  |  | T10607C |  | T10607C |  | T10607C |  | T10607C |  |
|  | G | T10643C |  |  |  | T10643C |  | T10643C |  | T10643C |  | T10643C |  | T10643C |  |
|  | G | T10646C | F566L |  |  |  |  | T10646C | F566L | T10646C | F566L | T10646C | F566L | T10646C | F566L |
|  | G | C10649T |  |  |  |  |  | C10649T |  | C10649T |  | C10649T |  | C10649T |  |
|  | G | T10664C | I572T |  |  |  |  | T10664C | I572T | T10664C | I572T | T10664C | I572T | T10664C | I572T |
| **Bangladesh clade_Specific** | G | C10670T |  |  |  |  |  | C10670T |  | C10670T |  | C10670T |  |  |  |
|  | G | T10677C | L577P |  |  |  |  | T10677C | L577P | T10677C | L577P | T10677C | L577P | T10677C | L577P |
|  | G | T10691C |  |  |  |  |  | T10691C |  | T10691C |  | T10691C |  | T10691C |  |
|  | G | C10709T |  |  |  |  |  | C10709T |  | C10709T |  | C10709T |  | C10709T |  |
|  | G | C10718T |  |  |  |  |  | C10718T |  | C10718T |  | C10718T |  | C10718T |  |
|  | G | A10721G |  |  |  |  |  |  |  |  |  |  |  | A10721G |  |
|  | G | G10730A |  |  |  | G10730A |  | G10730A |  | G10730A |  | G10730A |  | G10730A |  |
|  | L | T11450C |  |  |  | T11450C |  | T11450C |  | T11450C |  | T11450C |  | T11450C |  |
| **2023 India_Specific** | L | **C11528T** |  |  |  |  |  |  |  |  |  |  |  | **C11528T** |  |
| **Present in all clades** | L | G11534A |  | G11534A |  | G11534A |  | G11534A |  | G11534A |  | G11534A |  | G11534A |  |
| **Bangladesh clade_Specific** | L | G11624A |  |  |  |  |  | G11624A |  | G11624A |  | G11624A |  |  |  |
| **Bangladesh clade_Specific** | L | C11633T |  |  |  |  |  | C11633T |  | C11633T |  | C11633T |  |  |  |
|  | L | A11648T | I77V |  |  | A11648T | I77V | A11648T | I77V | A11648T | I77V | A11648T | I77V | A11648T | I77V |
| **Bangladesh clade_Specific** | L | C11690T | C91S |  |  |  |  | C11690T | C91S | C11690T | C91S | C11690T | C91S |  |  |
|  | L | T11698C | I94T |  |  | T11698C | I94T | T11698C | I94T | T11698C | I94T |  |  | T11698C | I94T |
|  | L | C11700T |  |  |  | C11700T |  | C11700T |  | C11700T |  | C11700T |  | C11700T |  |
|  | L | **A11752G** | **K112R** |  |  |  |  |  |  | **A11752G** | K112R |  |  |  |  |
|  | L | A11789G |  |  |  |  |  |  |  |  |  |  |  | A11789G |  |
|  | L | T11831C |  |  |  |  |  | T11831C |  |  |  | T11831C |  | T11831C |  |
|  | L | G12053A |  |  |  | G12053A |  | G12053A |  | G12053A |  | G12053A |  | G12053A |  |
|  | L | A12110G |  |  |  |  |  |  |  |  |  |  |  | A12110G |  |
|  | L | C12113T |  |  |  | C12113T |  |  |  |  |  |  |  | C12113T |  |
|  | L | G12164A |  |  |  |  |  |  |  | G12164A |  | G12164A |  | G12164A |  |
| **2018 India & 2021_India common** | L | A12167G |  |  |  |  |  |  |  |  |  |  |  | A12167G |  |
|  | L | C12185T |  |  |  |  |  |  |  |  |  |  |  | C12185T |  |
|  | L | **A12194G** |  |  |  | A12194G |  | A12194G |  | A12194G |  | A12194G |  | A12194G |  |
|  | L | **T12215C** |  |  |  |  |  |  |  | T12215C |  | T12215C |  |  |  |
|  | L | A12263G |  |  |  | A12263G |  | A12263G |  | A12263G |  | A12263G |  | A12263G |  |
|  | L | T12266C |  |  |  |  |  |  |  |  |  |  |  | T12266C |  |
|  | L | **G12320A** |  |  |  |  |  |  |  | G12320A |  | G12320A |  |  |  |
| **Bangladesh clade_Specific** | L | G12326A |  |  |  |  |  | G12326A |  | G12326A |  | G12326A |  |  |  |
|  | L | T12329C |  |  |  |  |  |  |  |  |  |  |  | T12329C |  |
|  | L | T12350C |  |  |  | T12350C |  | T12350C |  | T12350C |  | T12350C |  | T12350C |  |
|  | L | C12351T |  |  |  |  |  |  |  |  |  |  |  | C12351T |  |
|  | L | **T12384C** |  |  |  |  |  |  |  |  |  | T12384C |  |  |  |
|  | L | **T12407C** |  |  |  |  |  |  |  | T12407C |  |  |  |  |  |
|  | L | **T12428C** |  |  |  |  |  |  |  |  |  |  |  | T12428C |  |
|  | L | **A12443G** |  |  |  |  |  |  |  | A12443G |  |  |  |  |  |
|  | L | **A12542G** |  |  |  |  |  |  |  |  |  |  |  | A12542G |  |
|  | L | **G12563A** |  |  |  |  |  |  |  | G12563A |  | G12563A |  |  |  |
|  | L | **A12566G** |  |  |  |  |  |  |  | A12566G |  | A12566G |  |  |  |
|  | L | A12599G |  |  |  |  |  | A12599G |  | A12599G |  | A12599G |  | A12599G |  |
|  | L | **C12680T** |  |  |  |  |  |  |  | C12680T |  | C12680T |  | C12680T |  |
|  | L | A12695G |  |  |  | A12695G |  | A12695G |  | A12695G |  | A12695G |  | A12695G |  |
|  | L | T12843C |  |  |  |  |  |  |  |  |  |  |  | T12843C |  |
|  | L | A12875G |  |  |  |  |  | A12875G |  | A12875G |  | A12875G |  | A12875G |  |
|  | L | **A12968G** |  |  |  |  |  |  |  | A12968G |  | A12968G |  |  |  |
|  | L | G12980A |  |  |  | G12980A |  | G12980A |  | G12980A |  | G12980A |  | G12980A |  |
| **Bangladesh clade_Specific** | L | G13010A |  |  |  |  |  | G13010A |  | G13010A |  | G13010A |  |  |  |
|  | L | T13028C |  |  |  |  |  |  |  |  |  |  |  | T13028C |  |
|  | L | G13052A |  |  |  |  |  |  |  |  |  |  |  | G13052A |  |
|  | L | A13070T |  |  |  |  |  |  |  |  |  |  |  | A13070T |  |
|  | L | **T13071C** |  |  |  |  |  |  |  | T13071C |  | T13071C |  |  |  |
| **Bangladesh clade_Specific** | L | C13076T |  |  |  |  |  | C13076T |  | C13076T |  | C13076T |  |  |  |
|  | L | G13082A |  |  |  |  |  |  |  |  |  |  |  | G13082A |  |
|  | L | **G13175A** |  |  |  |  |  |  |  | G13175A |  | G13175A |  | G13175A |  |
|  | L | G13244A |  |  |  | G13244A |  |  |  |  |  |  |  | G13244A |  |
|  | L | C13262G |  |  |  |  |  |  |  |  |  |  |  | C13262G |  |
|  | L | C13286T | F623L |  |  |  |  |  |  |  |  |  |  | C13286T | F623L |
|  | L | T13307C | T630A |  |  | T13307C | T630A | T13307C | T630A | T13307C | T630A | T13307C | T630A | T13307C | T630A |
|  | L | **A13312G** | **N632S** |  |  |  |  |  |  | **A13312G** | **N632S** |  |  |  |  |
|  | L | **G13314A** | **V633M** |  |  |  |  |  |  | **G13314A** | V633M |  |  |  |  |
|  | L | **A13332G** | **N639D** |  |  |  |  |  |  | **A13332G** | **N632S** | **A13332G** | **N632S** |  |  |
|  | L | A13341T | N642Y |  |  |  |  | A13341T | N642Y | A13341T | N642Y | A13341T | N642Y | A13341T | N642Y |
|  | L | **T13343C** |  |  |  |  |  |  |  | T13343C |  |  |  |  |  |
|  | L | **C13411T** | **T665I** |  |  |  |  |  |  | **C13411T** | **T665I** | **C13411T** | **T665I** |  |  |
|  | L | **G13427A** |  |  |  |  |  |  |  | G13427A |  |  |  |  |  |
|  | L | C13454T |  |  |  |  |  |  |  |  |  |  |  | C13454T |  |
|  | L | T13490C |  |  |  | T13490C |  | T13490C |  | T13490C |  | T13490C |  | T13490C |  |
| **Bangladesh clade_Specific** | L | C13514T |  |  |  |  |  | C13514T |  | C13514T |  | C13514T |  |  |  |
|  | L | G13520T |  |  |  | G13520T |  | G13520T |  | G13520T |  | G13520T |  | G13520T |  |
| **2023 India_Specific** | L | **C13523A** |  |  |  |  |  |  |  |  |  |  |  | **C13523A** |  |
|  | L | G13532A |  |  |  | G13532A |  | G13532A |  | G13532A |  | G13532A |  | G13532A |  |
|  | L | T13541C |  |  |  | T13541C |  | T13541C |  | T13541C |  | T13541C |  | T13541C |  |
|  | L | A13547T |  |  |  |  |  |  |  | A13547T |  |  |  | A13547T |  |
|  | L | T13604C |  |  |  |  |  |  |  |  |  |  |  | T13604C |  |
| **Bangladesh clade_Specific** | L | C13721T |  |  |  |  |  | C13721T |  | C13721T |  | C13721T |  |  |  |
|  | L | A13760G |  |  |  |  |  |  |  |  |  |  |  | A13760G |  |
|  | L | A13764G | K783E |  |  | A13764G | K783E | A13764G | K783E | A13764G | K783E | A13764G | K783E | A13764G | K783E |
|  | L | **A13832G** |  |  |  |  |  |  |  |  |  |  |  | A13832G |  |
| **Bangladesh clade_Specific** | L | T13847C |  |  |  |  |  | T13847C |  | T13847C |  | T13847C |  |  |  |
|  | L | A13910G |  |  |  |  |  |  |  | A13910G |  | A13910G |  | A13910G |  |
|  | L | C13913T |  |  |  |  |  |  |  |  |  |  |  | C13913T |  |
|  | L | **T13949C** |  |  |  |  |  |  |  |  |  | T13949C |  |  |  |
|  | L | G13970A |  |  |  |  |  |  |  |  |  |  |  | G13970A |  |
|  | L | **G14012A** |  |  |  |  |  |  |  | G14012A |  |  |  |  |  |
|  | L | T14054C |  |  |  |  |  |  |  | T14054C |  | T14054C |  | T14054C |  |
|  | L | **C14066T** |  |  |  |  |  |  |  | C14066T |  |  |  |  |  |
|  | L | A14085G | I890V |  |  | A14085G | I890V | A14085G | I890V | A14085G | I890V | A14085G | I890V | A14085G | I890V |
|  | L | T14102C |  |  |  |  |  |  |  |  |  |  |  | T14102C |  |
| **Bangladesh clade_Specific** | L | A14138G |  |  |  |  |  | A14138G |  | A14138G |  | A14138G |  |  |  |
|  | L | T14171C |  |  |  | T14171C |  | T14171C |  | T14171C |  | T14171C |  | T14171C |  |
|  | L | C14172T |  |  |  | C14172T |  |  |  |  |  |  |  | C14172T |  |
|  | L | T14198C |  |  |  |  |  |  |  |  |  |  |  | T14198C |  |
|  | L | **A14219C** |  |  |  |  |  |  |  | A14219C |  |  |  |  |  |
|  | L | **A14249G** |  |  |  |  |  |  |  | A14249G |  |  |  |  |  |
|  | L | C14264T |  |  |  | C14264T |  | C14264T |  | C14264T |  | C14264T |  | C14264T |  |
|  | L | T14318C |  |  |  |  |  |  |  |  |  |  |  | T14318C |  |
|  | L | **C14334T** |  |  |  |  |  |  |  | C14334T |  | C14334T |  |  |  |
|  | L | A14390G |  |  |  |  |  |  |  |  |  |  |  | A14390G |  |
|  | L | G14489A |  |  |  | G14489A |  | G14489A |  | G14489A |  | G14489A |  | G14489A |  |
| **Present in all clades** | L | T14501C |  | T14501C |  | T14501C |  | T14501C |  | T14501C |  | T14501C |  | T14501C |  |
|  | L | A14528G |  |  |  |  |  |  |  |  |  |  |  | A14528G |  |
|  | L | **C14609T** |  |  |  |  |  |  |  | C14609T |  | C14609T |  |  |  |
|  | L | C14675T |  |  |  | C14675T |  | C14675T |  | C14675T |  | C14675T |  | C14675T |  |
| **Bangladesh clade_Specific** | L | T14720C |  |  |  |  |  | T14720C |  | T14720C |  | T14720C |  |  |  |
|  | L | C14771T |  |  |  |  |  |  |  |  |  |  |  | C14771T |  |
|  | L | **A14810G** |  |  |  |  |  |  |  | A14810G |  |  |  |  |  |
| **2018 India_Specific** | L | **C14846T** |  |  |  |  |  |  |  |  |  |  |  | **C14846T** |  |
|  | L | **T14847C** |  |  |  |  |  |  |  | T14847C |  | T14847C |  |  |  |
| **Bangladesh clade_Specific** | L | A14864G |  |  |  |  |  | A14864G |  | A14864G |  | A14864G |  |  |  |
|  | L | C14877A | L1154I |  |  | C14877A | L1154I | C14877A | L1154I | C14877A | L1154I | C14877A | L1154I | C14877A | L1154I |
|  | L | T14909C |  |  |  |  |  |  |  |  |  |  |  | T14909C |  |
|  | L | **C14934T** |  |  |  |  |  |  |  | C14934T |  |  |  | C14934T |  |
|  | L | C14939T |  |  |  | C14939T |  | C14939T |  | C14939T |  | C14939T |  | C14939T |  |
| **Bangladesh clade_Specific** | L | T15020C |  |  |  |  |  | T15020C |  | T15020C |  | T15020C |  |  |  |
|  | L | G15041T | A1208D |  |  | G15041T | A1208D | G15041T | A1208D | G15041T | A1208D | G15041T | A1208D | G15041T | A1208D |
|  | L | A15080C |  |  |  |  |  | A15080C |  | A15080C |  | A15080C |  | A15080C |  |
|  | L | G15086A |  |  |  |  |  | G15086A |  | G15086A |  | G15086A |  | G15086A |  |
|  | L | G15095A |  |  |  |  |  |  |  |  |  |  |  | G15095A |  |
|  | L | G15113A |  |  |  |  |  | G15113A |  |  |  | G15113A |  | G15113A |  |
|  | L | **G15122A** |  |  |  |  |  |  |  | G15122A |  |  |  |  |  |
|  | L | **C15125T** |  |  |  |  |  |  |  |  |  |  |  | C15125T |  |
|  | L | G15188A |  |  |  | G15188A |  | G15188A |  | G15188A |  | G15188A |  | G15188A |  |
| **Bangladesh clade_Specific** | L | G15202A | R1262K |  |  |  |  | G15202A | R1262K | G15202A | R1262K | G15202A | R1262K |  |  |
|  | L | G15206A |  |  |  |  |  |  |  |  |  |  |  | G15206A |  |
| **Bangladesh clade_Specific** | L | A15281G | K1288R |  |  |  |  | A15281G | K1288R | A15281G | K1288R | A15281G | K1288R |  |  |
|  | L | G15344A |  |  |  | G15344A |  | G15344A |  | G15344A |  | G15344A |  | G15344A |  |
|  | L | C15347T |  |  |  | C15347T |  | C15347T |  | C15347T |  | C15347T |  | C15347T |  |
|  | L | G15389A |  |  |  |  |  |  |  |  |  |  |  | G15389A |  |
|  | L | **G15392A** |  |  |  |  |  |  |  | G15392A |  |  |  |  |  |
|  | L | T15414C |  |  |  |  |  |  |  |  |  |  |  | T15414C |  |
| **2019_India_specific** | L | C15428T |  |  |  |  |  |  |  |  |  |  |  | C15428T |  |
|  | L | **C15437T** |  |  |  |  |  |  |  |  |  | C15437T |  |  |  |
|  | L | C15455T |  |  |  | C15455T |  |  |  |  |  |  |  | C15455T |  |
|  | L | **T15485C** |  |  |  |  |  |  |  |  |  | T15485C |  |  |  |
|  | L | A15512G |  |  |  | A15512G |  | A15512G |  | A15512G |  | A15512G |  | A15512G |  |
|  | L | T15518C |  |  |  |  |  |  |  |  |  |  |  | T15518C |  |
|  | L | C15542T |  |  |  | C15542T |  | C15542T |  | C15542T |  | C15542T |  | C15542T |  |
|  | L | A15569G |  |  |  |  |  | A15569G |  | A15569G |  | A15569G |  | A15569G |  |
| **2018 India_Specific** | L | **T15590C** |  |  |  |  |  |  |  |  |  |  |  | **T15590C** |  |
|  | L | **G15644A** |  |  |  |  |  |  |  | G15644A |  |  |  |  |  |
|  | L | T15677C |  |  |  |  |  |  |  |  |  |  |  | T15677C |  |
|  | L | C15686T |  |  |  | C15686T |  | C15686T |  | C15686T |  | C15686T |  | C15686T |  |
|  | L | **A15734G** |  |  |  | A15734G |  | A15734G |  | A15734G |  | A15734G |  |  |  |
|  | L | G15743A |  |  |  |  |  | G15743A |  | G15743A |  | G15743A |  | G15743A |  |
| **Bangladesh clade_Specific** | L | A15767G |  |  |  |  |  | A15767G |  | A15767G |  | A15767G |  |  |  |
| **Bangladesh clade_Specific** | L | G15770T |  |  |  |  |  | G15770T |  | G15770T |  | G15770T |  |  |  |
|  | L | C15845A |  |  |  | C15845A |  | C15845A |  | C15845A |  | C15845A |  | C15845A |  |
| **Bangladesh clade_Specific** | L | A15887G |  |  |  |  |  | A15887G |  | A15887G |  | A15887G |  |  |  |
|  | L | C15923T |  |  |  |  |  |  |  | C15923T |  |  |  | C15923T |  |
| **2023 India_Specific** | L | **C16032T** | **P1539S** |  |  |  |  |  |  |  |  |  |  | **C16032T** | **P1539S** |
| **2023 India_Specific** | L | **T16039C** | **L1541P** |  |  |  |  |  |  |  |  |  |  | **T16039C** | **L1541P** |
|  | L | T16106C |  |  |  | T16106C |  |  |  | T16106C |  | T16106C |  | T16106C |  |
|  | L | C16211T |  |  |  |  |  | C16211T |  | C16211T |  |  |  | C16211T |  |
|  | L | G16214A |  |  |  |  |  |  |  |  |  |  |  | G16214A |  |
|  | L | T16298C |  |  |  | T16298C |  | T16298C |  | T16298C |  | T16298C |  | T16298C |  |
|  | L | C16331T |  |  |  |  |  |  |  |  |  |  |  | C16331T |  |
| **Present in all clades** | L | C16351A | S1645Y | C16351A | S1645Y | C16351A | S1645Y | C16351A | S1645Y | C16351A | S1645Y | C16351A | S1645Y | C16351A | S1645Y |
| **Bangladesh clade_Specific** | L | T16391C | S1658N |  |  |  |  | T16391C | S1658N | T16391C | S1658N | T16391C | S1658N |  |  |
| **Bangladesh clade_Specific** | L | C16514T |  |  |  |  |  | C16514T |  |  |  | C16514T |  |  |  |
|  | L | T16520C |  |  |  |  |  |  |  |  |  |  |  | T16520C |  |
| **2023 India_Specific** | L | **G16538A** | **V1707M** |  |  |  |  |  |  |  |  |  |  | **G16538A** | **V1707M** |
|  | L | **G16574A** |  |  |  | G16574A |  |  |  | G16574A |  |  |  |  |  |
| **Bangladesh clade_Specific** | L | A16583G | V1722A |  |  |  |  | A16583G | V1722A | A16583G | V1722A | A16583G | V1722A |  |  |
|  | L | **A16595G** |  |  |  |  |  |  |  | A16595G |  |  |  |  |  |
|  | L | A16727G |  |  |  |  |  |  |  |  |  |  |  | A16727G |  |
|  | L | A16763G |  |  |  |  |  |  |  |  |  |  |  | A16763G |  |
|  | L | A16790G | A1791S |  |  | A16790G | A1791S | A16790G | A1791S | A16790G | A1791S | A16790G | A1791S | A16790G | A1791S |
| **Malaysia_Specific** | L | A16794G | K1793E | A16794G | K1793E |  |  |  |  |  |  |  |  |  |  |
|  | L | **T16817C** |  |  |  |  |  |  |  | T16817C |  |  |  |  |  |
|  | L | **G16823A** |  |  |  |  |  |  |  | G16823A |  |  |  |  |  |
|  | L | T16871A |  |  |  | T16871A |  | T16871A |  | T16871A |  | T16871A |  | T16871A |  |
|  | L | T16884C |  |  |  |  |  |  |  |  |  |  |  | T16884C |  |
|  | L | G16901A |  |  |  | G16901A |  | G16901A |  | G16901A |  | G16901A |  | G16901A |  |
|  | L | G16919A |  |  |  |  |  |  |  |  |  |  |  | G16919A |  |
|  | L | G16922A |  |  |  | G16922A |  | G16922A |  | G16922A |  | G16922A |  | G16922A |  |
|  | L | **A16931G** |  |  |  |  |  |  |  |  |  |  |  | A16931G |  |
| **2018 India_Specific** | L | **T16940C** |  |  |  |  |  |  |  |  |  |  |  | **T16940C** |  |
|  | L | **T16986C** |  |  |  |  |  |  |  | T16986C |  |  |  |  |  |
| **2019_India_specific** | L | C17009T |  |  |  |  |  |  |  |  |  |  |  | C17009T |  |
|  | L | T17030C | D1871E |  |  |  |  |  |  |  |  |  |  | T17030C | D1871E |
| **Bangladesh clade_Specific** | L | A17057G |  |  |  |  |  | A17057G |  | A17057G |  | A17057G |  |  |  |
|  | L | C17133T |  |  |  | C17133T |  | C17133T |  | C17133T |  | C17133T |  | C17133T |  |
|  | L | A17144G |  |  |  | A17144G |  | A17144G |  | A17144G |  | A17144G |  | A17144G |  |
|  | L | C17165T |  |  |  |  |  |  |  | C17165T |  | C17165T |  |  |  |
| **2019_India_specific** | L | A17266G | E1950G |  |  |  |  |  |  |  |  |  |  | A17266G | E1950G |
|  | L | T17384C |  |  |  |  |  |  |  | T17384C |  |  |  |  |  |
|  | L | C17405T |  |  |  | C17405T |  | C17405T |  | C17405T |  | C17405T |  | C17405T |  |
| **2023 India_Specific** | L | **T17424C** | **F2003L** |  |  |  |  |  |  |  |  |  |  | **T17424C** | **F2003L** |
| **2023 India_Specific** | L | **C17427A** | **P2004T** |  |  |  |  |  |  |  |  |  |  | **C17427A** | **P2004T** |
|  | L | A17432G |  |  |  | A17432G |  | A17432G |  | A17432G |  | A17432G |  | A17432G |  |
|  | L | T17441C |  |  |  |  |  | T17441C |  | T17441C |  | T17441C |  | T17441C |  |
|  | L | A17456G |  |  |  | A17456G |  | A17456G |  | A17456G |  | A17456G |  | A17456G |  |
| **2018 India & 2021_India common** | L | T17472C |  |  |  |  |  |  |  |  |  |  |  | T17472C |  |
|  | L | G17477A |  |  |  | G17477A |  | G17477A |  | G17477A |  | G17477A |  | G17477A |  |
| **Bangladesh clade_Specific** | L | G17483A |  |  |  |  |  | G17483A |  | G17483A |  | G17483A |  |  |  |
|  | L | T17525C |  |  |  |  |  |  |  |  |  |  |  | T17525C |  |
| **Bangladesh clade_Specific** | L | A17526G | N2037D |  |  |  |  | A17526G | N2037D | A17526G | N2037D | A17526G | N2037D |  |  |
| **2023 India_Specific** | L | **C17534T** | **H2039N** |  |  |  |  |  |  |  |  |  |  | **C17534T** | **H2039N** |
|  | L | A17555G |  |  |  | A17555G |  | A17555G |  | A17555G |  | A17555G |  | A17555G |  |
| **Bangladesh clade_Specific** | L | A17576G |  |  |  |  |  | A17576G |  | A17576G |  | A17576G |  |  |  |
|  | L | A17834G |  |  |  |  |  |  |  |  |  |  |  | A17834G |  |
|  | L | T17892C | C2159R |  |  | T17892C | C2159R | T17892C | C2159R | T17892C | C2159R | T17892C | C2159R | T17892C | C2159R |
| **Present in all clades** | L | A17903G |  | A17903G |  | A17903G |  | A17903G |  | A17903G |  | A17903G |  | A17903G |  |
|  | L | C17948T |  |  |  | C17948T |  | C17948T |  | C17948T |  | C17948T |  | C17948T |  |
|  | L | T17957C |  |  |  | T17957C |  | T17957C |  | T17957C |  | T17957C |  | T17957C |  |
|  | L | T17960C |  |  |  | T17960C |  | T17960C |  | T17960C |  | T17960C |  | T17960C |  |
|  | L | C17966T | I2183T |  |  | C17966T | I2183T | C17966T | I2183T | C17966T | I2183T | C17966T | I2183T | C17966T | I2183T |
|  | L | G18011A |  |  |  | G18011A |  | G18011A |  | G18011A |  | G18011A |  | G18011A |  |
|  | L | A18064G | N2216S |  |  | A18064G | N2216S | A18064G | N2216S | A18064G | N2216S | A18064G | N2216S | A18064G | N2216S |
|  | L | A18074G | K2219E |  |  |  |  |  |  |  |  | A18074G | K2219E | A18074G | K2219E |
|  | L | A18077G |  |  |  | A18077G |  | A18077G |  | A18077G |  | A18077G |  | A18077G |  |
|  | L | T18143C |  |  |  | T18143C |  | T18143C |  | T18143C |  | T18143C |  | T18143C |  |

References:

de Campos GM, Cella E, Kashima S, Alcântara LCJ, Sampaio SC, Elias MC, Giovanetti M, Slavov SN. Updated Insights into the Phylogenetics, Phylodynamics, and Genetic Diversity of Nipah Virus (NiV). Viruses. 2024 Jan 24;16(2):171. doi: 10.3390/v16020171. PMID: 38399947; PMCID: PMC10892031.
