# Supplementary material for: Pandemic potential of the Nipah virus and public health strategies adopted during outbreaks: Lessons from Kerala, India
Source: PLOS Glob Public Health. 2024 Dec 19;4(12):e0003926. doi: 10.1371/journal.pgph.0003926 (PMC11658523; doi:10.1371/journal.pgph.0003926)
Supplement: S2 Table — (DOCX) [file pgph.0003926.s002.docx]

**S2 Table:**

**Comparison chart of the Nipah virus vaccines under clinical trials**

| **Feature** | **HeV-sG-V** | **PHV02** | **mRNA-1215** | **ChAdOx1 NiV** |
| --- | --- | --- | --- | --- |
| **Type** | Subunit Vaccine | Live attenuated | mRNA Vaccine | Viral Vector Vaccine |
| **Developer** | Emergent BioSolutions/  Auro vaccines | Public Health Vaccines, LLC | Moderna | University of Oxford |
| **Target Antigen** | Soluble G glycoprotein (sG) | Whole virus (attenuated) | Encodes NiV surface proteins | Encodes NiV surface proteins |
| **Administration** | Intramuscular | Intramuscular | Intramuscular | Intramuscular |
| **Doses Required** | 2 doses | Single dose | 2 doses | Single dose |
| **Current Phase** | Phase I | Phase I | Phase I | Phase I/II |
| **Storage Requirements** | Standard refrigeration (2-8°C) | Standard refrigeration (2-8°C) | Ultra-low freezer (-20°C to -80°C) | Standard refrigeration (2-8°C) |
| **Potential Advantages** | Established platform | Potential for single-dose immunity | Rapid development and production | Established platform |
| **Potential Limitations** | Requires adjuvant | Potential reversion to virulence | Cold chain requirements | Previous ChAdOx1 trials mixed results |
| **Clinical trial findings** | Safe and immunogenic and the two doses have the potential for both reactive outbreak control and preventative use. | Currently into Phase-I trial | Not available (trial recruiting) | **Pre-clinical trial data:** A single vaccination with ChAdOx1 NiV protects African green monkeys against lethal disease induced by Nipah virus inoculation. A robust humoral and cellular response was detected starting 14 days post initial vaccination.  **Clinical trial-** Phase-I data not available yet |

References:

Frenck, Robert and Naficy, Abdi and Feser, Jodi and Egan, Michael and Chen, Tracy and Dickey, Michelle and Eldridge, John H. and Sciotto-Brown, Susan and Hermida, Luz and Leyva-Grado,, V. H. and Promeneur, Dominique H. and Luckay, Amara and Medina, Herbert and Lazaro, Glorie-Grace H. and Patel, Nairuti and Naqvi, Tahira and Gast, Christopher and Mercer, Laina D. and Raine, Mike and Andi-Lolo, Indah and Innis, Bruce L. and Aponte, John and Hamm, Stefan and Rathi, Niraj, Safety and Immunogenicity of a Nipah Virus Vaccine (Hev-Sg-V) in Adults: A Single-Centre, Randomised, Observer-Blind, Placebo-Controlled, Phase 1 Study. Available at SSRN: <https://ssrn.com/abstract=4845156> or [http://dx.doi.org/10.2139/ssrn.4845156](https://dx.doi.org/10.2139/ssrn.4845156)

van Doremalen, N., Avanzato, V.A., Goldin, K. *et al.* ChAdOx1 NiV vaccination protects against lethal Nipah Bangladesh virus infection in African green monkeys. *npj Vaccines* **7**, 171 (2022). https://doi.org/10.1038/s41541-022-00592-9
